# Protein fortification with mealworm (*Tenebrio molitor L*.) powder: Effect on textural, microbiological, nutritional and sensory features of bread

**DOI:** 10.1371/journal.pone.0211747

**Published:** 2019-02-01

**Authors:** Andrea Roncolini, Vesna Milanović, Federica Cardinali, Andrea Osimani, Cristiana Garofalo, Riccardo Sabbatini, Francesca Clementi, Marina Pasquini, Massimo Mozzon, Roberta Foligni, Nadia Raffaelli, Federica Zamporlini, Gabriele Minazzato, Maria Federica Trombetta, Anse Van Buitenen, Leen Van Campenhout, Lucia Aquilanti

**Affiliations:** 1 Dipartimento di Scienze Agrarie, Alimentari ed Ambientali, Università Politecnica delle Marche, via Brecce Bianche, Ancona, Italy; 2 KU Leuven, Department of Microbial and Molecular Systems (M2S), Faculty of Engineering Technology, Lab4Food, Technology Campus Geel, Geel, Belgium; 3 KU Leuven, Leuven Food Science and Nutrition Rese Centre (LFoRCe), Leuven, Belgium; Tulane University, UNITED STATES

## Abstract

In the present study, inclusion of mealworm (*Tenebrio molitor* L.) powder into bread doughs at 5 and 10% substitution level of soft wheat (*Triticum aestivum* L.) flour was tested to produce protein fortified breads. The addition of mealworm powder (MP) did not negatively affect the technological features of either doughs or breads. All the tested doughs showed the same leavening ability, whereas breads containing 5% MP showed the highest specific volume and the lowest firmness. An enrichment in protein content was observed in experimental breads where the highest values for this parameter were recorded in breads containing 10% MP. Breads fortified with 10% MP also exhibited a significant increase in the content of free amino acids, and especially in the following essential amino acids: tyrosine, methionine, isoleucine, and leucine. By contrast, no differences in nutritional quality of lipids were seen between fortified and control breads. Results of sensory analyses revealed that protein fortification of bread with MP significantly affected bread texture and overall liking, as well as crust colour, depending on the substitution level. Overall, proof of concept was provided for the inclusion of MP into bread doughs started with different leavening agents (sourdough and/or baker’s yeast), at 5 or 10% substitution level of soft wheat flour. Based on the Technology Readiness Level (TRL) scale, the proposed bread making technology can be situated at level 4 (validation in laboratory environment), thus suggesting that the production of breads with MP might easily be scaled up at industrial level. However, potential spoilage and safety issues that need to be further considered were highlighted.

## Introduction

Bread is a staple food throughout Europe and western countries; it is obtained from the baking of a leavened dough commonly prepared with wheat flour, water, and a leavening agent, with or without the addition of salt (sodium chloride) and other ingredients (e.g. malt, enzymes, animal fats, oils, hydrogenated fats, margarine, sugars, milk powder, bread improvers, stabilizers, etc.). Among the leavening agents, different alternatives can be used, including: (i) chemicals (e.g. sodium bicarbonate); (ii) baker's yeast, which essentially consists of *Saccharomyces cerevisiae* biomass; and (iii) sourdough. The latter consists of a mixture of flour and water spontaneously fermented by (or inoculated with) a mixed population of lactic acid bacteria (LAB) and yeasts and propagated by back-slopping [1]. The use of the sourdough leads to the production of bread with enhanced sensory and nutritional traits as well as an extended shelf-life [2]. Western white bread produced with wheat flour is usually rich in carbohydrates that represent about 50% of dry weight (w/w), whereas its protein content is generally very low, with average values generally comprised between 6 and 8% (w/w) [3, 4], thus rendering bread as an ideal candidate for protein fortification. The World Health Organization (WHO) and the Food and Agricultural Organization of the United Nations (FAO) have defined fortification as "*the practice of deliberately increasing the content of an essential micronutrient*, *ie*. *vitamins and minerals (including trace elements) in a food irrespective of whether the nutrients were originally in the food before processing or not*, *so as to improve the nutritional quality of the food supply and to provide a public health benefit with minimal risk to health*”.

The role of high value proteins in the diet of living beings is crucial, since they are pivotal in nutrient synthesis and degradation as well as in metabolic functions. Proteins are also essential to maintain muscle mass and strength, especially in elderly people [5].

Protein-energy malnutrition and protein security are challenges on a global level, especially in low-income countries in the presence of a generalized low food intake and other adverse environmental factors [6]. In high-income countries, including European ones, the recommended daily intake of proteins of 0.66 g per kilogram of body weight is generally met by adults of both sexes consuming mixed diets [7]. However, even in these countries, suboptimal protein intake can be found in older adults [8], where it has been related to sarcopenia, a condition characterized by loss of skeletal muscle mass and function [9]. Hence, the formulation of attractive protein-enriched foods represents a challenge for the modern food industry. Besides to increasing the quantity of protein intake, food fortification may also potentially improve the quality of proteins supplied with the diet e.g. adding more essential amino acids to the diet. With this goal in mind, the search for new protein sources is constantly facing new challenges in Europe [5] as well as worldwide [10]. To date, a number of nutrition interventions to boost protein intake through the consumption of bread have been explored, including, among others, the use of cumin and caraway seed by-product flours [11], whey and soy protein hydrolysates [5], and even insect powders [3, 12, 13].

Protein ingredients used for fortification of bread and other leavened baked goods can alter sensory features of the end products, with a consequent reduction of palatability and overall consumer acceptability, as it has recently been found for muffins [14] and bread enriched with cricket (*Acheta domesticus*) powder [3].

Regarding the powders from edible insects, both scientific research and food industries recognize the high potential offered by these alternative protein ingredients for food and feed uses. Edible insects are part of the diet of at least 2 billion people all over Asia, Africa, and South America. In Europe and the United States, consumers’ interest in insect-based foods is now slowly increasing. The European Food Safety Authority (EFSA) has recently proposed the following list of insect species with the greatest potential to be used as food and feed in the EU: *Acheta domesticus*, *Achroia grisella*, *Alphitobius diaperinus*, *Bombyx mori*, *Galleria mellonella*, *Gryllodes sigillatus*, *Hermetia illucens*, *Locusta migratoria migratorioides*, *Musca domestica*, *Schistocerca americana*, *Tenebrio molitor*, and *Zophobas atratus* [15].

Besides to being rich in high-quality proteins and essential amino acids, edible insects are also characterized by a high content in lipids, micronutrients, and vitamins [16]. Moreover, compared with traditional livestock, rearing of edible insects presents numerous advantages, since insects (i) cause low emissions of greenhouse gases and ammonia; (ii) undergo rapid multiplication, with more efficient feed conversion; and (iii) require a relatively limited rearing space [17].

Considering the high potential of edible insects as a novel ingredient for protein fortification of bread [3, 12, 13], the present study was aimed at: (i) exploring the use of mealworm (*Tenebrio molitor* L.) powder for fortification of soft wheat (*Triticum aestivum* L.) breads with high protein content and appealing sensory properties; (ii) evaluating the effects of increasing amounts of mealworm powder on the textural, microbiological, nutritional and sensory characteristics of the fortified breads; (iii) evaluating the effect of two different leavening agents (baker’s yeast and sourdough) on the above listed attributes. To this end, experimental bread loaves produced using different blends of wheat flour and mealworm powder and leavened using either baker’s yeast or a combination of baker’s yeast and sourdough were subjected to chemical, microbiological, textural, and sensory analyses as well as to overall liking evaluation by a panel of untrained panelists.

## Material and methods

### Flour and insect powder

Soft wheat (*T*. *aestivum* L.) flour (WF), classified as type 00 and used for both technological evaluation of blends and bread making, was provided by a local mill (Molino Stacchiotti, Ancona, Italy), whereas mealworm (*T*. *molitor* L.) powder (MP), containing the whole lipid fraction, was purchased from Kreca Ento-Food BV (Ermelo, The Netherlands). Samples of MP were packaged in 100 g plastic bags each and stored at ambient temperature until use. No information was available on the rearing conditions of mealworms or hygiene practices applied during powder production, storage and transport before buying.

### Rheological evaluation of wheat flour and flour blends

Farinograph (UNI 10790:1999 standard method), alveograph (UNI EN ISO 27971:2015 standard method), and microvisco-amylograph (UNI 10872:2000 standard method) tests were carried out to evaluate the rheological properties of the WF and blends of WF and MP (5% and 10% MP). No rheological analyses were performed on MP, given the assumption that the latter is not a bread-making ingredient as such.

### Sourdough production with selected lactic acid bacteria strains

The sourdough used as a leavening agent was produced through the inoculation of five LAB strains, namely: *Lactobacillus fermentum* PB162, *Lactobacillus plantarum* PB11, *Lactobacillus plantarum* PB24, *Lactobacillus sanfranciscensis* PB276, and *Lactobacillus sanfranciscensis* PB223. The selected strains, belonging to the culture collection of the Dipartimento di Scienze Agrarie, Alimentari ed Ambientali (D3A) (Università Politecnica delle Marche), were previously isolated from wheat sourdoughs produced in the Marche region (central Italy) and characterized for their technological traits (acidification, amylase activity, starch hydrolysis and lactate, acetate and CO_2_ production) by Osimani et al. [18].

The frozen stored cultures were grown as reported by Osimani et al. [3]. Briefly, *L*. *fermentum* PB162, *L*. *plantarum* PB11 and *L*. *plantarum* PB24 were cultivated on modified De Man, Rogosa and Sharp (MRS) agar (Oxoid, Basingtoke, UK) added with 1% maltose (w/v) and 5% fresh yeast extract (v/v) prepared according to Gobbetti et al. [19], whereas *L*. *sanfranciscensis* PB276 and *L*. *sanfranciscensis* PB223 were cultivated on Sourdough Bacteria (SDB) medium modified in accordance with Vogel et al. [20].

Sourdough was produced as previously reported by Osimani et al. [3]. Briefly, selected LAB pure cultures were separately cultivated in modified MRS broth at 30°C for 12 h. The obtained broth cultures were centrifuged at 4,000 rpm for 5 min, washed, and resuspended in sterile tap water [21]. The pool of selected LAB strains, wheat flour and sterilized water were admixed to reach dough yield 167 (60 g/100 g wheat flour plus 40 g/100 g water). The obtained dough, at an initial LAB concentration of 8 log cfu g^-1^, was left to ferment at 30°C for 16 h in order to obtain the mature sourdough (SD) used for bread making trials.

### Dough composition and bread-making

Two different amounts of MP were used in addition to WF in order to produce experimental bread loaves, whereas the sole WF was used to produce control breads. In more detail, MP was used at a substitution level of WF equal to 5 and 10% (w/w) regardless of the sourdough WF content. In bread added with sourdough, the flour:sourdough ratio was set at 3:1. In all the dough formulations, water was added in order to reach a dough yield 160 whereas baker’s yeast was added at a concentration of 2% (Table 1).

**Table 1:** Formulations of doughs (D) obtained with the use of wheat flour (W) or different blends of wheat flour and 5% or 10% mealworm powder (M) admixed with baker’s yeast as leavening agents and sourdough (S).

Ingredients	Experimental doughs					
	WD	WDS	MD_5_	MDS_5_	MD_10_	MDS_10_
Wheat flour (g/100 g dough)	61.25	52.06	58.19	49.46	55.13	46.86
Mealworm powder (g/100 g dough)	n.a.	n.a.	3.06	2.60	6.13	5.21
Water (g/100 g dough)	36.75	30.63	36.75	30.63	36.75	30.63
Sourdough (g/100 g dough)	n.a.	15.31	n.a.	15.31	n.a.	15.31
Baker’s Yeast (g/100 g dough)	2.00	2.00	2.00	2.00	2.00	2.00
Obtained bread (B) loaves	WB	WBS	MB_5_	MBS_5_	MB_10_	MBS_10_

n.a. not added

The leavening performance of the doughs (MD_5_, MDS_5_, MD_10_, MDS_10_, WD, and WDS), carried out at 30°C, was evaluated using graduated glass cylinders (2 L) as previously described [22]. Briefly, each dough was prepared as described in Table 1 and manually kneaded for about 10 minutes up until a proper gluten development was reached. In order to estimate the volume increase of doughs, they were placed in graduated glass cylinders and poured with 40 mL paraffin at their top to prevent moisture loss and drying. The volume of each dough (in mL) was recorded soon after placement in the cylinders (t_0_) and after a 2 hour fermentation (t_2_). The leavening was calculated using the following formula: [(V_2_—V_0_)/V_0_] x 100, where V_0_ was the volume at t_0_ and V_2_ was the volume after fermentation. The results were expressed as means ± standard deviations of duplicate experiments.

The breads (B) (Table 1) made with MD_5_, MDS_5_, MD_10_, MDS_10_, WD, and WDS were obtained through a one-step fermentation process (30°C for 1 h) followed by 1 h oven baking at 200°C as previously described by Osimani et al. [3]. Core temperature of baked breads was measured soon after baking using a portable thermometer (Checktemp 1—HI 98509, Hannah Instruments, Padova, Italy). Bread-making trials were carried out in duplicate.

### Determination of pH and total titratable acidity (TTA)

Direct potentiometric pH assessment of sourdoughs and doughs was carried out with a pH meter (HI2031, Hanna Instruments, Padova, Italy) equipped with a solid electrode (HI2031, Hanna Instruments, Padova, Italy).

Total titratable acidity (TTA) was assessed in accordance with the method already described by Minervini et al. [23]. Briefly, 10 g of each sourdough or dough was homogenized in 90 mL distilled water and the suspensions were then titrated with 0.1 N NaOH to a final pH of 8.5. The TTA results were expressed as the amount of NaOH (mL) used. Both pH and TTA analyses were carried out in duplicate and the results reported as mean ± standard deviation.

### Analysis of bread firmness

Bread firmness was assessed as previously described by Osimani et al. [3]. Briefly, experimental breads were centre sliced (25 mm thick) and bread slices positioned on the sample table under the load cell of a CT3-4500 texture analyzer (Brookfiled Engineering Laboratories Inc., Middleboro MA, USA) equipped with a 36 mm diameter bread probe (mod. TA-AACC36). The probe compressed the crumb to a 40% compression limit at a speed of 100 mm min^-^_1_. A 4500 g load cell was used. All measurements were carried out in duplicate and the results reported as mean ± standard deviation.

### Microbiological analyses

MP, WF, sourdough, doughs, and breads underwent plate counting of LAB, yeasts and aerobic bacterial spores, depending on the type of sample. LAB occurring in MP, WF, sourdoughs (t_0_ and t_16h_) and doughs (t_1h_) were counted on both MRS (Oxoid) and SDB agar supplemented with cycloheximide (0.2 g L^-1^) (Merck KGaA, Darmstadt, Germany) to inhibit the growth of yeasts as previously described by Osimani et al. [18]. Yeasts occurring in MP, WF, sourdoughs (t_0_ and t_16h_), and doughs (t_1h_) were enumerated on Rose Bengal Agar (Oxoid) supplemented with chloramphenicol (0.1 g L^-1^) (Oxoid) as already described by Cardinali et al. [24]. Aerobic bacterial spores occurring in all samples except for sourdoughs (t_0_ and t_16h_) and doughs (t_0_) were counted on Standard Plate Count Agar (Oxoid) as previously described [25].

Finally, counts of *Bacillus cereus* and *Clostridium perfringens* in all bread samples were carried out in accordance with UNI EN ISO 7932:2005 and ISO 7937:2004 standard methods, respectively.

The results of viable counting were expressed as mean of log colony forming units (cfu) per gram of sample ± standard deviation.

### PCR-DGGE analysis

Plates of aerobic bacterial spores showing a number of colonies comprised between 30 and 300 were used for bulk formation prior to Polymerase Chain Reaction–Denaturing Gradient Gel Electrophoresis (PCR-DGGE) analysis, as previously described [3]. When colonies grown on plates were below 30, bulk cells were collected from the first dilution plates that showed any growth.

The DNA was extracted from bulk colonies as described by Osimani et al. [26]. The quantity and the purity of the extracted DNA was assessed by Nanodrop ND 1000 (Thermo Fisher Scientific, Wilmington, DE, USA) and standardised to a concentration of 25 ng μL^-1^ for further analysis. Two μL (about 50 ng) of each DNA extract was amplified by PCR in a My Cycler Thermal Cycler (BioRad Laboratories, Hercules, CA, USA) using the universal prokaryotic primers 338F and 518r [27] and PCR conditions described by Osimani et al. [26]. As proposed by Ampe et al. [28], the forward primer 338F was added with a GC clamp, as required for the subsequent DGGE analysis. The DGGE analysis as well as the sequencing and identification of the excised DGGE bands were performed as detailed by Osimani et al. [29]. Only the sequences showing more than 97% of similarity with the sequences deposited in the GenBank database (http://www.ncbi.nlm.nih.gov) were clearly assigned to a species or a genus.

### Proximate composition

Chemical analyses were carried out to assess the content of the following parameters: dry matter, moisture, protein, fat, ash, nitrogen free extract (NFE). Energy values were determined in accordance with the Atwater system [30]. The reference analytical methods used are those previously reported by Osimani et al. [3]. All the chemical analyses were carried out in duplicate. The results were expressed as % (w/w) and reported as mean values ± standard deviations.

### Amino acid analyses

Protein hydrolysis was performed with 6 M HCl, in the presence of 3 mM sarcosine as an internal standard, at 110°C for 24 h, under vacuum. Derivatization of amino acids with ortophthalaldehyde (OPA) and 9-fluorenylmethyloxycarbonyl (FMOC) was carried out as described by Henderson et al. [31]. Derivatized amino acids were separated by high performance liquid chromatography (HPLC) on a Zorbax Eclipse Plus C18 column, 5 μm, 250 x 4.6 mm (Agilent Technologies, Santa Clara, CA, USA), as described by Henderson & Brooks [32] with the following modifications: a 20-min equilibration of the column was included in the elution conditions, and temperature was maintained at 55°C. Absorbance was monitored at 338 nm and 262 nm for OPA- and FMOC-derivatized amino acids, respectively.

For tryptophan determination, 5-mg samples were incubated with 1.0 ml 5 N NaOH, at 120°C for 24 h, under vacuum. Hydrolysates were diluted 10-fold with water and neutralized to pH 6.0 using a concentrated HCl solution. Samples were filtered through 0.2 μm filters, centrifuged, and injected into a Luna C18 column, 5 μm, 250 x 4.6 mm (Phenomenex, Torrance, CA, USA). Elution was performed with 25 mM sodium acetate, pH 6.0, 15% (v/v) acetonitrile for 12 min. Flow rate was maintained at 0.9 mL min^-1^ and the temperature was fixed at 18°C. Absorbance was monitored at 280 nm.

Extraction of free amino acids was performed by vigorously vortexing 30-mg aliquots of samples in 0.2 ml 0.1 N HCl, containing 0.25 mM sarcosine. After centrifugation at 20,000 x *g* for 10 min at 4°C, pellets were washed with additional 0.2 ml 0.1 N HCl and centrifuged as above. Supernatants were pooled and filtered through a 0.45 μm filter. Derivatization and analysis was performed as described above.

### Total fatty acids analysis

Fatty acid methyl esters (FAMEs) were prepared by direct acid-catalysed transesterification of dried samples (WF, MP, and breads) and were analysed by a Trace 1300 gas chromatograph (Thermo Fisher Scientific, Waltham, MA USA) equipped with a column TG-Polar, 60 m length × 0.25 mm i.d., 0.2 μm film thickness (Thermo Fisher Scientific). Details about derivatisation procedure and chromatographic conditions have previously been reported by Osimani et al. [33].

### Colour analysis

The bread colour was assessed according to the CIELAB system (CIE, 1986); accordingly, the lightness (L*), redness (a*), and yellowness (b*) of the crust and crumb bread samples were recorded using Minolta CR 200 with D65 illuminant, 10° viewing angle and an aperture of 30 mm. The instrument was calibrated using white standard coordinates. Results were expressed as mean ± standard deviation of triplicate analyses.

### Sensory analysis

Overall liking for fortified and control breads was assessed as previously described [3]. Briefly, 9 untrained panellists, including 5 females and 4 males with age comprised between 23–60 years, regularly consuming wheat bread were chosen. Liking of experimental breads was ranked with a 9-point hedonic scale, where 1 and 9 correspond to extreme disliking and liking, respectively. Data were expressed as mean ± standard deviation of three independent experiments. All assessors involved in the sensory analysis were informed about the aim of the study and provided their informed written consent to the D3A. Moreover, the need for approval of the sensory analysis was prospectively waived by the Ethical Committee of the Università Politecnica delle Marche.

### Statistical analysis

The Tukey-Kramer’s Honest Significant Difference (HSD) test (level of significance 0.05) was used to evaluate differences inside groups (blends of WF and MP, breads), by one-way analysis of variance (ANOVA). Experimental data were explored by Principal Component Analysis (PCA); normalization was used as data pre-treatment procedure. The software JMP Version 11.0.0 (SAS Institute Inc., Cary, NC) was used to carry out all tests.

## Results and discussion

### Technological characterization

The results of the chemical characterization of the raw materials are reported in Table 2.

**Table 2:** Chemical traits of the wheat flour (WF) and mealworm powder (MP) used for bread-making.

Parameters	Samples	
	WF	MP
Dry matter (%)	88.59 ± 0.06	93.88 ± 0.14
Water (%)	11.40 ± 0.06	6.12 ± 0.14
Protein (%)	11.38 ± 0.01	51.71 ± 0.71
Fat (%)	0.88 ± 0.05	27.69 ± 0.11
Ash (%)	0.48 ± 0.01	3.65 ± 0.07
NFE (%)	71.72 ± 0.17	5.15 ± 0.74
Energy (kcal 100 g^-1^)	348.61 ± 0.83	488.06 ± 0.66

Means ± standard deviations of duplicate independent experiments are shown.

In more detail, regarding the WF, the mean values recorded for all of the parameters were in the range of those previously reported by different authors for this baking ingredient [13, 3, 34].

Concerning MP, high protein (51.71 ± 0.71%) and fat (27.69 ± 0.11%) mean values were measured. These values are in line with the values reported on the website of the supplier, 54.1 and 29.4%, respectively. The recorded values were also in the range of those previously reported by González et al. [13] in mealworm powder used for bread making and similar to those detected by Osimani et al. [33] in whole dried mealworms.

The rheological characterization of WF and the two blends of WF and MP (namely, MP_5_ and MP_10_) is reported in Table 3.

**Table 3:** Rheological characterization of the wheat flour (WF) and two blends of wheat flour and mealworm powder (MP_5_ and MP_10_) used for bread-making.

Parameters	Samples		
	WF	MP_5_	MP_10_
P/L	0.65 ± 0.02^c^	0.96 ± 0.01^b^	1.4 ± 0.02^a^
W (10^−4^ J)	260.0 ± 2.0^a^	222.0 ± 1.0^b^	176.0 ± 1.0^c^
Consistency (BU)	497.0 ± 1.0^a^	492.0 ± 2.0^a^	493.0 ± 1.0^a^
Water absorption (%)	59.7 ± 0.20^a^	59.3 ± 0.20^a^	58.9 ± 0.20^a^
Dough development time (min)	14.0 ± 0.10^a^	12.7 ± 0.05^a^	11.2 ± 0.20^a^
Dough stability (min)	26.0 ± 0.05^a^	21.8 ± 0.20^a^	22.0 ± 0.10^a^
Mixing tolerance index after 10 min (BU)	12.0 ± 2.0^a^	11.0 ± 1.0^a^	7.0 ± 1.0^b^
Mixing tolerance index after 12 min (BU)	19.0 ± 1.0^c^	37.0 ± 2.0^a^	28.0 ± 1.0^b^
Maximum viscosity (BU)	1402.0 ± 3.0^a^	1345.0 ± 1.0^b^	1189.0 ± 2.0^c^
Falling number (sec)	420.0 ± 1.0^a^	420.0 ± 2.0^a^	415.0 ± 2.0^a^

WF, 100% wheat flour; MP_5_, blend containing 5% mealworm powder and 95% wheat flour; MP_10_, blend containing 10% mealworm powder and 90% wheat flour.

P/L (tenacity:extensibility ratio), where P represents the maximum overpressure needed to blow the dough bubble and L the average abscissa at bubble rupture.

W expresses the strength of the flour or blend

BU: Brabender Units.

Means ± standard deviations of duplicate independent experiments are shown.

Within each row, means followed by different letters (a, b, c) are significantly different (P < 0.05).

As expected, the addition of MP to WF affected the ratio between tenacity and extensibility, expressed as P/L, where P expresses the maximum over-pressure needed to blow a dough bubble and L the average abscissa at bubble rupture [35]. As it emerges from experimental data, the higher the amount of MP added, the higher the ratio P/L, leading to the statistically significantly highest value (1.4 ± 0.02) for the blend MP_10_. It is noteworthy that all the blends were within the range of P/L values appropriate for baking (> 0.5), as suggested by Graça et al. [36]. The results confirmed the trends already reported by Osimani et al. [3] in a similar study that evaluated the use of cricket (*A*. *domesticus*) powder in bread making.

Moreover, the addition of MP negatively affected the strength of the dough, expressed as W. In more detail, the higher the amount of MP added, the lower the W value was. Indeed, the measured strength of the doughs dropped from 260.0 ± 2.0 in WF to 176.0 ± 1.0 in MP_10_. It is likely that the reduced amount of total gluten in the blends, due to the addition of MP, influenced the gluten network formation in the doughs during mixing, thus progressively lowering their strength, as previously suggested by Pasqualone et al. [37].

The consistency of the dough, expressed in Brabender Units (BU), was not affected by the addition of MP. Similarly, no significant differences regarding water absorption percentage for WF and the two tested blends were observed. In bread making, the amount of added water represents a pivotal factor that allows the distribution of the dough ingredients, their hydration and the formation of the gluten network, thus increasing the bread yield [36]. The consistency and water absorption mean values recorded in the present study confirm the trends reported by Osimani et al. [3], although in the latter study, different substitution levels of insect powder (10 and 30%) were assayed. It is likely that the proteins from the MP have replaced in some manner the wheat gluten proteins that were reduced by insect powder addition; this hypothesis is supported by rheological data collected by Graça et al. [36] who investigated the impact of a further protein-rich gluten-free ingredient (*Chlorella vulgaris*) on the rheology of wheat flour dough and bread texture. It is noteworthy that González et al. [13] observed a reduction in water absorption in WF dough containing 5% MP. Further research is needed to understand the chemical interactions among insect powder proteins and wheat dough.

Both the dough development time and dough stability were not affected by MP addition. These results are similar to those reported by Osimani et al. [3] for the same parameters assessed in blends of WF and cricket powder at 10%, whereas the addition of 30% of the same insect powder led to a higher dough development time and lower dough stability [3]. Hence, it is likely that at low substitution levels (e.g., 5 or 10%), the addition of insect powder does not strongly affect these two parameters.

The use of a farinograph allowed defining the mixing tolerance of WF and blends of WF and MP (MP_5_ and MP_10_) used for bread-making. This parameter is the difference in BU from the top of the curve at the peak time to the top of the curve 5 minutes after the peak is reached. A higher value means that the flour breaks down faster after reaching full development. Usually, a mixing tolerance of 30 BU or less is considered very good to excellent, whereas a value greater than 50 BU highlights less tolerance and more frequent difficulties during kneading of the dough. Regarding the mixing tolerance after 10 minutes, the lowest mean value (7.0 ± 1.0 min) was recorded for the blend MP_10_, whereas no differences between WF and MP_5_ were observed. The mixing tolerance after 12 minutes was the highest for MP_5_ (37.0 ± 2.0 min) and the lowest for WF (19.0 ± 1.0 min). These results do not agree with those reported by Osimani et al. [3] for doughs containing cricket powder. It is likely that different amounts and types of insect powder added to WF can differently affect the mixing tolerance of the resulting dough. Moreover, the relationship between dough mixing properties and gluten protein composition has already been elucidated, as well as the influence of polymeric protein fraction changes occurring during mixing [38]. Further studies are needed to understand the complex modifications occurring in the protein-starch dough mixing properties after insect powder addition.

Dough viscosity can be influenced by a number of factors, including amylase activity and starch quality [39], as well as fat content [40]. In the samples analyzed in this study, the maximum viscosity was negatively influenced by the addition of MP. Indeed, the highest and the lowest mean values were recorded for WF (1402.0 ± 3.0 BU) and the MP_10_ blend (1189.0 ± 2.0 BU), respectively. These results are in accordance with those previously reported by Osimani et al. [3], thus suggesting that fat contained in insect powders does not compensate the shortage of starch and amylose of these unconventional baking ingredients.

The falling number represents a valid indicator of α-amylase activity [39]. In the present study, no differences in the mean falling number values were recorded among WF and the two tested blends containing MP. This is most likely due to the low substitution level of WF with MP.

**Fig 1:**
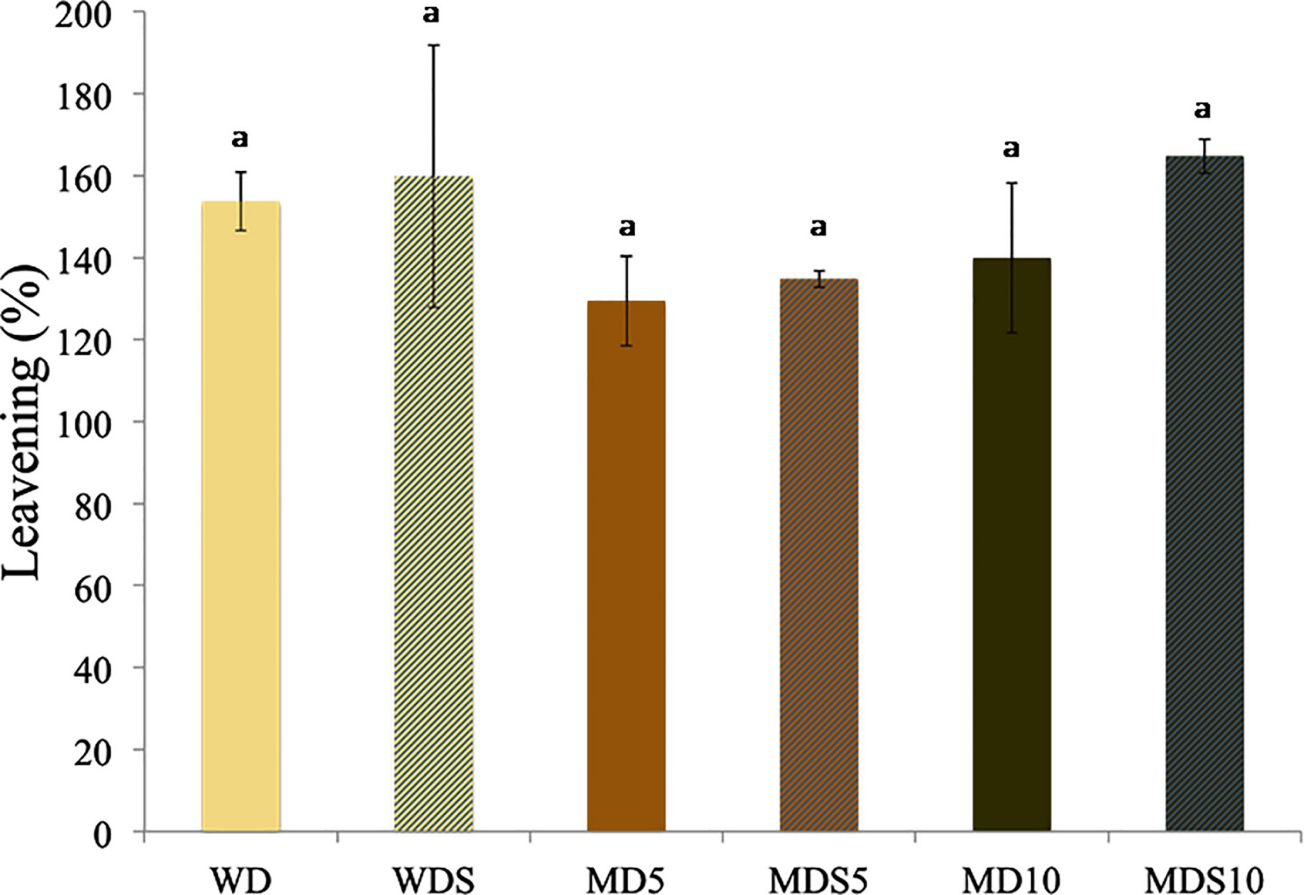
Leavening of the doughs (D) prepared with the different blends of wheat flour (W) and mealworm powder (M) and admixed with baker’s yeast as leavening agent and sourdough (S). Samples are codified as reported in Table 1. Means ± standard deviations of triplicate independent experiments are shown. Means with different superscripts are significantly different (*P* > 0.05).

As shown in Fig 1, all doughs tested exhibited the same leavening ability, irrespective of the use of baker’s yeast alone or baker’s yeast in combination with sourdough as leavening agents or the amount of added MP.

**Fig 2:**
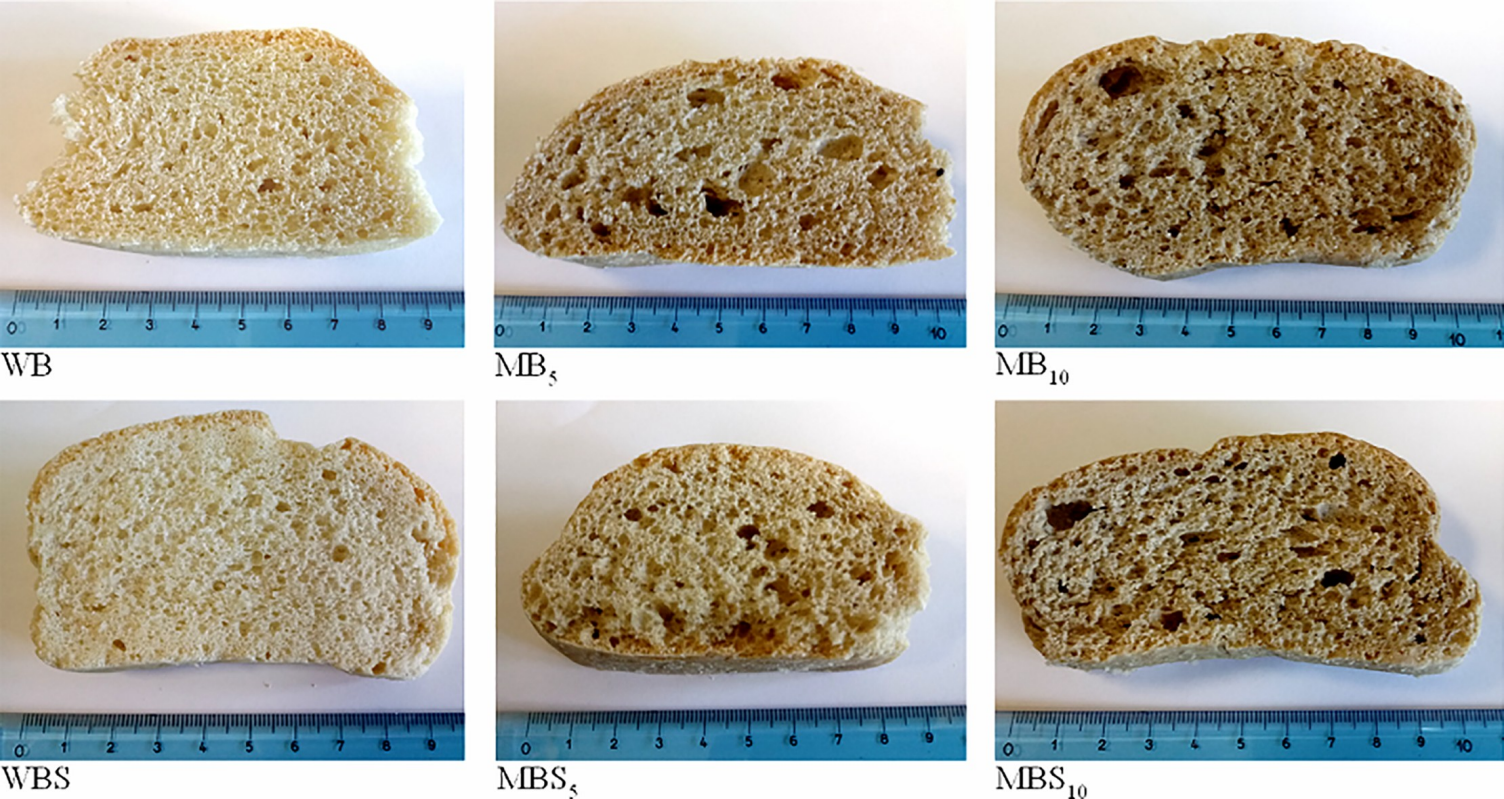
Slices obtained from the loaves of experimental breads. Samples are codified as reported in Table 1.

**Fig 3:**
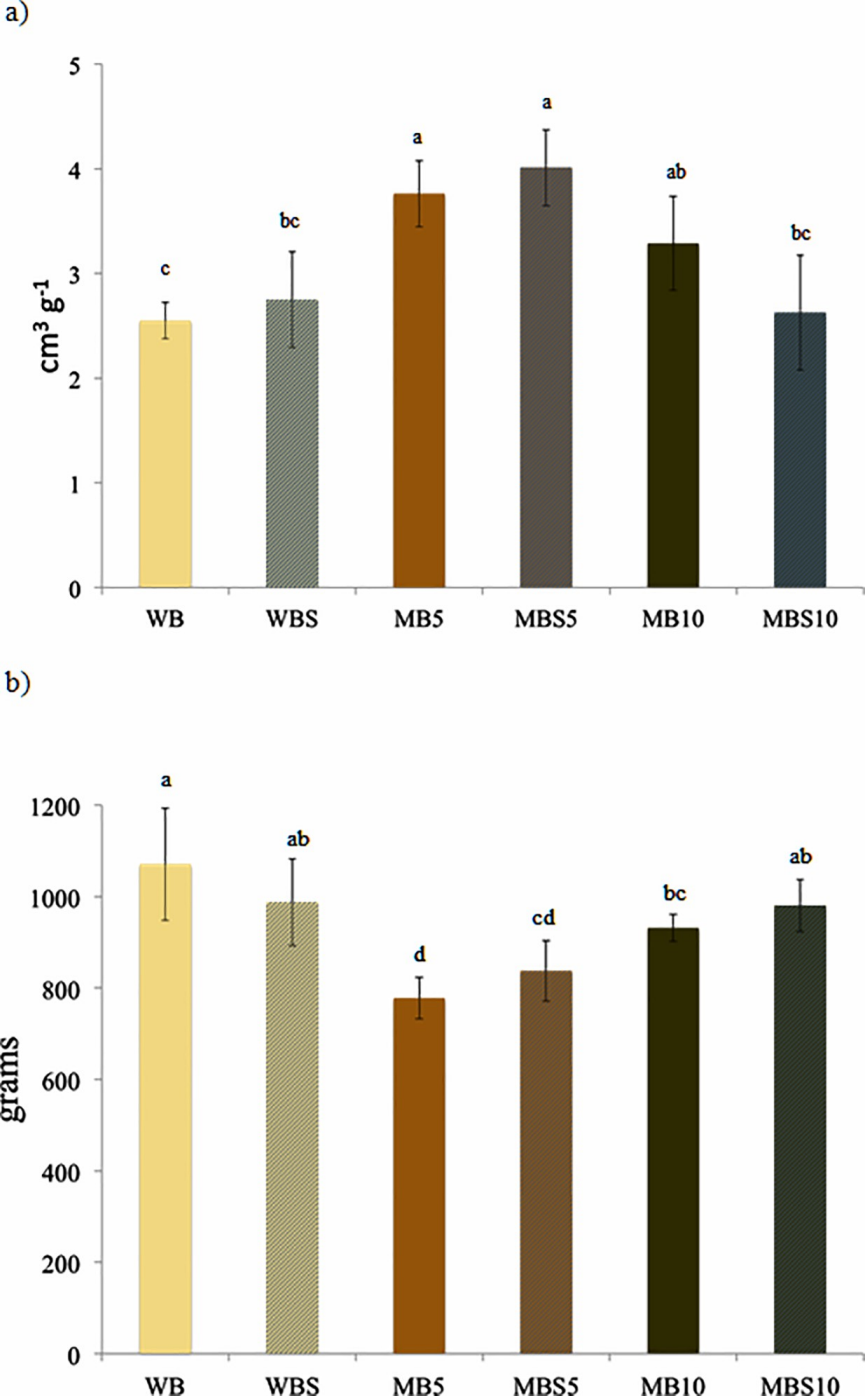
**Specific volume (panel a) and firmness (panel b) of bread (B) loaves prepared with the different blends of wheat flour (W) and mealworm powder (M) and admixed with baker’s yeast as leavening agent and sourdough (S).** Samples are codified as reported in Table 1. Means ± standard deviations of triplicate independent experiments are shown. Means with different superscripts are significantly different (*P* > 0.05).

The recorded values ranged between 129.38 ± 11.00% for MD_5_ and 164.71 ± 4.16% for MDS_10_. These results are in agreement with the trends reported by Osimani et al. [3] for the leavening ability of wheat doughs enriched with cricket powder. Fig 2 shows the slices obtained from the experimental bread loaves, whereas the results of the specific volume and firmness measurements are shown in Fig 3.

Regarding the specific volume, significantly higher values than for the other conditions were recorded for MB_5_ and MBS_5_ with mean values of 3.76 ± 0.38 and 4.02 ± 0.36 cm^3^ g^-1^, respectively. The lowest mean value was recorded for WB (2.55 ± 0.17 cm^3^ g^-1^). Regarding the bread firmness, the highest mean value was measured in WB (1071.06 ± 122.77 grams), whereas MB_5_ exhibited the lowest (777.87 ± 45.00 grams). It is noteworthy that de Oliveira et al. [12] reported a linear correlation between specific volume and bread firmness in their study regarding bread prepared with insect powder, which can also explain the bread loaves with the lowest specific volume exhibiting the highest hardness in our study. In the present study, the addition of MP yielded an enhancement of the specific volume and a softer bread compared with the control breads. These findings can be likely ascribed to the fat fraction of the added MP; it is known that in bread making fat is often incorporated as an antistaling agent and volume improver [41]. As reported by Pareyt, [42], added fats plasticize and lubricate the dough, with an increase in air incorporation during mixing. In such a system, lipid crystals complex with the gluten network, thus leading to dough stabilization and strengthening. Moreover, fats, being adsorbed at the gas cell-dough interface, increase gas retention during leavening. During baking, the melting of fats stabilizes the expanding gas cells [42].

The results of the proximate analyses of the experimental breads produced with the use of WF (namely, WB and WBS) or blends containing WF and MP (namely, MB_5_, MBS_5_, MB_10_, and MBS_10_) are reported in Table 4.

**Table 4:** Proximate composition of bread (B) produced with wheat flour (W) or different blend of wheat flour and mealworm powder (M) and admixed with sourdough (S) and baker’s yeast as leavening agents.

Parameters	Reference method	Samples					
		WB	WBS	MB_5_	MBS_5_	MB_10_	MBS_10_
Dry matter (%)	AOAC, 950.46	69.98 ± 0.73^a^	69.93 ± 1.34^a^	70.80 ± 1.53^a^	68.35 ± 1.37^a^	69.05 ± 2.07^a^	69.83 ± 3.67^a^
Moisture (%)	AOAC, 1999	30.01 ± 0.73^a^	30.07 ± 1.34^a^	29.19 ± 1.53^a^	31.65 ± 1.37^a^	30.94 ± 2.07^a^	30.16 ± 3.67^a^
Protein (%)	AOAC, 981.10	8.91 ± 0.23^d^	9.21 ± 0.24^d^	10.54 ± 0.30^b^	9.80 ± 0.30^c^	11.55 ± 0.33^a^	11.62 ± 0.08^a^
Fat (%)	AOAC, 991.36	0.09 ± 0.02^d^	0.15 ± 0.10^d^	0.48 ± 0.02^c^	0.40 ± 0.05^c^	1.11 ± 0.04^a^	0.92 ± 0.06^b^
Ash (%)	AOAC, 920.153	0.49 ± 0.13^bc^	0.43 ± 0.04^c^	0.53 ± 0.02^abc^	0.50 ± 0.01^abc^	0.64 ± 0.01^a^	0.62 ± 0.04^ab^
NFE (%)	Osimani et al., 2017	60.5 ± 1. 35^a^	60.13 ± 1.40^a^	59.24 ± 0.32^ab^	57.64 ± 0.10^b^	55.74 ± 1.14^c^	56.66 ± 2.19^bc^
Energy (kcal 100 g^-1^)	Merrill and Watt, 1973	273.72 ± 4.33^a^	272.71 ± 4.70^a^	279.27 ± 4.29^a^	268.70 ± 3.49^a^	274.26 ± 11.36^a^	277.07 ± 14.78^a^

AOAC, Association of Official Analytical Chemists International.

Samples are codified as reported in Table 1.

Means ± standard deviations of duplicate independent measurements are shown.

Within each row, means followed by different letters (a, b, c, d) are significantly different (*P* < 0.05).

No significant differences in terms of dry matter or moisture were observed among the analyzed samples. A notable enrichment in both protein and fat content was observed in bread containing MP, irrespective of the use of baker’s yeast alone or in combination with sourdough. In particular, the enrichment in the protein content encourages the exploitation of edible insect powders in bread making.

Regarding the protein content, the lowest mean values were observed for WB (8.91 ± 0.23%) and WBS (9.21 ± 0.24%), whereas the highest values were recorded for MB_10_ (11.55 ± 0.33%) and MBS_10_ (11.62 ± 0.08%). Both breads produced with MP_5_ (namely, MB_5_ and MBS_5_) exhibited intermediate values. Those data are in agreement with the trends observed by González et al. [13] for bread produced with 5% MP and, more generally, with those reported by Osimani et al. [3] and de Oliveira et al. [12] for breads enriched with other insect powders.

As expected, MP addition produced an increase in protein content as a function of the increasing rate of MP fortification in the bread formulations. More specifically, average protein content in breads fortified with 10% MP increased by approximately 27% compared to the control breads, whereas breads fortified with 5% MP showed an average increase of 12%.

In the present study, protein content of the analyzed samples was determined with the Kjeldahl method, which is still recognized as the official method for protein determination by the Association of Official Analytical Chemists (AOAC) International. As reported by Jonas-Levi and Martinez [43], in edible insects the protein content is represented by easy-to-digest proteins, inaccessible proteins, chitin, and other N-containing molecules (e.g., nucleic acids). As for the content of chitin and inaccessible proteins, the reported values are extremely variable, largely depending on the stage of insect development (adults vs. larvae) [43]. In this specific food matrix, the use of the Kjeldahl method, which is not able to distinguish among the different sources of nitrogen, can lead to an overestimation of protein content. Based on these premises, the adopted conversion factors to date available for the Kjeldahl method should be revised and further studies should be carried out to develop specific N conservation factors for the determination of crude proteins in edible insects.

As for fat content, the same trend as observed for protein was revealed. The lowest fat mean values were reported for WB (0.09 ± 0.02%) and WBS (0.15 ± 0.10%), whereas MB_10_ (1.11± 0.04%) and MBS_10_ (0.92 ± 0.06%) exhibited the highest ones. These results are consistent with those reported by González et al. [13] and Osimani et al. [3]. Modification of the fat content (i.e., defattening) of insect powder to obtain a better-balanced enriched bread is recommended [12].

Regarding ash content, the minimal mean value was recorded in WB (0.49 ± 0.13%), whereas the maximum was observed in MB_10_ (0.64 ± 0.01%).

Finally, no statistically significant differences in the energy content of the analyzed samples were observed.

### Microbiological characterization

As requested by Regulation (EU) No. 2015/2283 [44] regarding novel foods, the commercialization of foods containing insects and their parts must be subjected to the European Commission (EC) authorization once that EFSA has performed risk assessments on their safety upon request by the EC. In such a context, the applicant may be requested by the EFSA or by the EC to provide additional information for the purposes of risk assessment or risk management, respectively. It is known that edible insects and insect-based ingredients harbor complex microbial communities that originate from their intestinal tract or from the rearing and processing environments. Among the reported species, saprophytic, spoilage or potentially pathogenic microorganisms can likely be present [3, 25, 29, 33, 45, 46, 47, 48, 49, 50, 51, 52].

In the present study, microbiological characterization was performed to evaluate the viable counts of pro-technological (LAB and yeasts) and aerobic bacterial spores in the raw materials, sourdough, and doughs. Moreover, the presence of endospores in the final breads was assessed, together with the occurrence of the following human pathogens: *C*. *perfringens* and *B*. *cereus*. The results of viable counting are reported in Table 5.

**Table 5:** Results of microbiological characterization of wheat flour (W), mealworm powder (M), sourdough (S_0_) (before fermentation), doughs (D) (before fermentation and after 1h fermentation) and mixed with baker’s yeast as leavening agents and sourdough (S), and experimental breads (B).

Samples		LAB on MRS	LAB on SBD	Yeasts	Aerobic spores	pH	TTA
		(log cfu g^-1^)					
Insect powder	MP	3.46 ± 0.65	3.81 ± 0.81	0.22 ± 0.53	2.81 ± 0.56	n.d.	n.d.
Wheat flour	WF	1.45 ± 1.21	1.44 ± 0.93	0.84 ± 0.99	1.04 ± 1.26	n.d.	n.d.
Sourdough (0 h)	S_0_	7.34 ± 0.15	7.33 ± 0.16	<1	n.d.	5.85 ± 0.02	1.05 ± 0.22
Sourdough (16 h)	S_16_	9.16 ± 0.08	9.16 ± 0.12	<1	n.d.	3.69 ± 0.10	9.33 ± 1.04
Dough (0 h)	MD_10_	n.d.	n.d.	n.d.	n.d.	5.88 ± 0.10	3.65 ± 0.50
	MDS_10_	n.d.	n.d.	n.d.	n.d.	5.39 ± 0.06	4.41 ± 0.72
	MD_5_	n.d.	n.d.	n.d.	n.d.	5.70 ± 0.12	2.30 ± 0.28
	MDS_5_	n.d.	n.d.	n.d.	n.d.	5.15 ± 0.06	3.92 ± 0.14
	WD	n.d.	n.d.	n.d.	n.d.	5.60 ± 0.04	1.71 ± 0.19
	WDS	n.d.	n.d.	n.d.	n.d.	5.01 ± 0.06	2.82 ± 0.82
Dough (1 h)	MD_10_	4.59 ± 0.32	4.56 ± 0.35	7.91 ± 0.54	1.35 ± 0.99	5.82 ± 0.01	3.57 ± 0.80
	MDS_10_	8.34 ± 0.09	8.39 ± 0.14	8.00 ± 0.04	<1	5.24 ± 0.04	4.34 ± 0.65
	MD_5_	3.58 ± 0.50	3.58 ± 0.69	7.67 ± 0.40	<1	5.57 ± 0.05	2.02 ± 0.17
	MDS_5_	8.39 ± 0.04	8.22 ± 0.33	7.89 ± 0.20	<1	5.05 ± 0.04	3.88 ± 0.59
	WD	3.95 ± 0.91	3.89 ± 0.83	7.81 ± 0.16	1.04 ± 1.65	5.47 ± 0.01	1.93 ± 0.32
	WDS	8.37 ± 0.12	8.39 ± 0.17	6.92 ± 0.47	0.25 ± 0.46	4.83 ± 0.05	2.84 ± 0.92
Bread	MB_10_	n.d.	n.d.	n.d.	<1	6.24 ± 0.11	2.28 ± 0.16
	MBS_10_	n.d.	n.d.	n.d.	<1	5.61 ± 0.02	3.72 ± 0.50
	MB_5_	n.d.	n.d.	n.d.	<1	6.01 ± 0.03	2.80 ± 0.31
	MBS_5_	n.d.	n.d.	n.d.	<1	5.57 ± 0.05	2.68 ± 0.42
	WB	n.d.	n.d.	n.d.	0.38 ± 0.52	6.15 ± 0.09	1.64 ± 0.45
	WBS	n.d.	n.d.	n.d.	0.58 ± 1.07	5.52 ± 0.18	2.63 ± 0.46

Samples are codified as reported in Table 1.

TTA was expressed as mL of 0.1 N NaOH.

n.d. not determined.

Regarding MP, the counts of LAB on both MRS and SDB media were in the range of 3 to 4 log cfu g^-1^, whereas the aerobic bacterial spores were present at 2.81 ± 0.56 log cfu g^-1^. Low counts of yeasts were recorded, with a mean value of 0.22 ± 0.53 log cfu g^-1^. The counts of LAB were slightly greater than those reported by Osimani et al. [25, 47] in whole dried edible mealworms, whereas the numbers of yeast counts were consistent with the values reported in the studies cited above. Finally, the counts of aerobic bacterial spores were in the range of those reported by Klunder et al. [53] in crushed and boiled mealworms.

The microbiological characterization of WF revealed LAB loads in the range of 1 to 2 log cfu g^-1^. LAB are naturally present at low levels in cereal-based matrices. The results of viable counts performed on WF were consistent with those reported by Alfonzo et al. [54] for samples of semolina collected in Italy. In the present study, counts of 0.84 ± 0.99 log cfu g^-1^ for fungi were detected in WF, whereas, in the same matrix the aerobic bacterial spore counts exhibited a mean value of 1.04 ± 1.26 log cfu g^-1^. Regarding bacterial spore-formers, different authors have already reported the occurrence of *Bacillus* strains in wheat semolina and grains, thus suggesting a potential spoilage or pathogenic effect in the final bread [55, 56, 57].

Microbial viable counts performed on sourdough at both t_0_ and after 16 h of fermentation evidenced high loads of LAB in both MRS and SDB, with mean counts that reached 9.16 ± 0.08 log cfu g^-1^, thus attesting the appropriateness of the inoculum and the growth of the inoculated selected strains in the dough during incubation. The LAB activity was also reflected by the pH reduction from 5.85 ± 0.02 (t_0_) to 3.69 ± 0.01 (t_16_) and the TTA increase from 1.05 ± 0.22 (t_0_) to 9.33 ± 1.04 (t_16_) mL of 0.1 N NaOH. The recorded TTA values are caused by the production of organic acids by sourdough LAB [1]. Yeast counts performed in sourdough at both t_0_ and after 16 h of fermentation revealed counts below 1 log cfu g^-1^.

The bread doughs produced with the sole use of baker’s yeast as leavening agent (namely, WD, MD_5_, and MD_10_) analyzed after 1 h of fermentation exhibited LAB counts between 3.58 ± 0.05 log cfu g^-1^ (MD_5_ on MRS) and 4.59 ± 0.32 log cfu g^-1^ (MD_10_ on MRS). The recorded values were slightly lower than those reported by Osimani et al. [3] for doughs containing WF or a blend of WF and cricket powder. As expected, doughs produced with the use of both baker’s yeast and sourdough as leavening agents (namely, WDS, MDS_5_, and MDS_10_) exhibited LAB counts as high as 8.39 ± 0.17 log cfu g^-1^, thus showing the growth of sourdough LAB in the final doughs. The LAB counts in doughs containing sourdough were in accordance with those reported by Osimani et al. [3] for doughs inoculated with the same amount of 16-h-fermented sourdough. Regarding yeast counts, the doughs exhibited mean values between 6.92 ± 0.47 log cfu g^-1^ (WDS) and 8.00 ± 0.04 log cfu g^-1^ (MDS_10_), thus illustrating the effects of the baker’s yeast.

Finally, the spore counts just before baking exhibited relatively low mean values that ranged between less than 1 and 1.35 ± 0.99 log cfu g^-1^. Moreover, the lowest values were found in the bread crumb analyzed soon after baking, with viable counts between less than 1 and 0.58 ± 1.07 log cfu g^-1^.

Overall, the occurrence of aerobic bacterial spores in bread crumb can be explained by their baking heat resistance. In this study, the core temperature of bread loaves ranged between 96.65 ± 0.86 and 97.83 ± 0.59°C, thus likely leading to an incomplete inactivation of the spores.

As reported by different authors, edible insects are natural carriers of bacterial spores that can be transferred to the food matrix when edible insects are used as food ingredients [3, 33, 49, 58, 59]. Hence, to obtain a more detailed picture of the viable spores occurring in the analyzed samples, bulk cells collected from PCA agar plates with spore counts greater than 1 log cfu g^-1^ were subjected to PCR-DGGE analysis. Since the late 1990s, PCR-DGGE has been widely applied to the study of food and environmental microbial communities, and it still represents one of the most powerful molecular tools to investigate the microbiota of food matrices [60].

**Fig 4:**
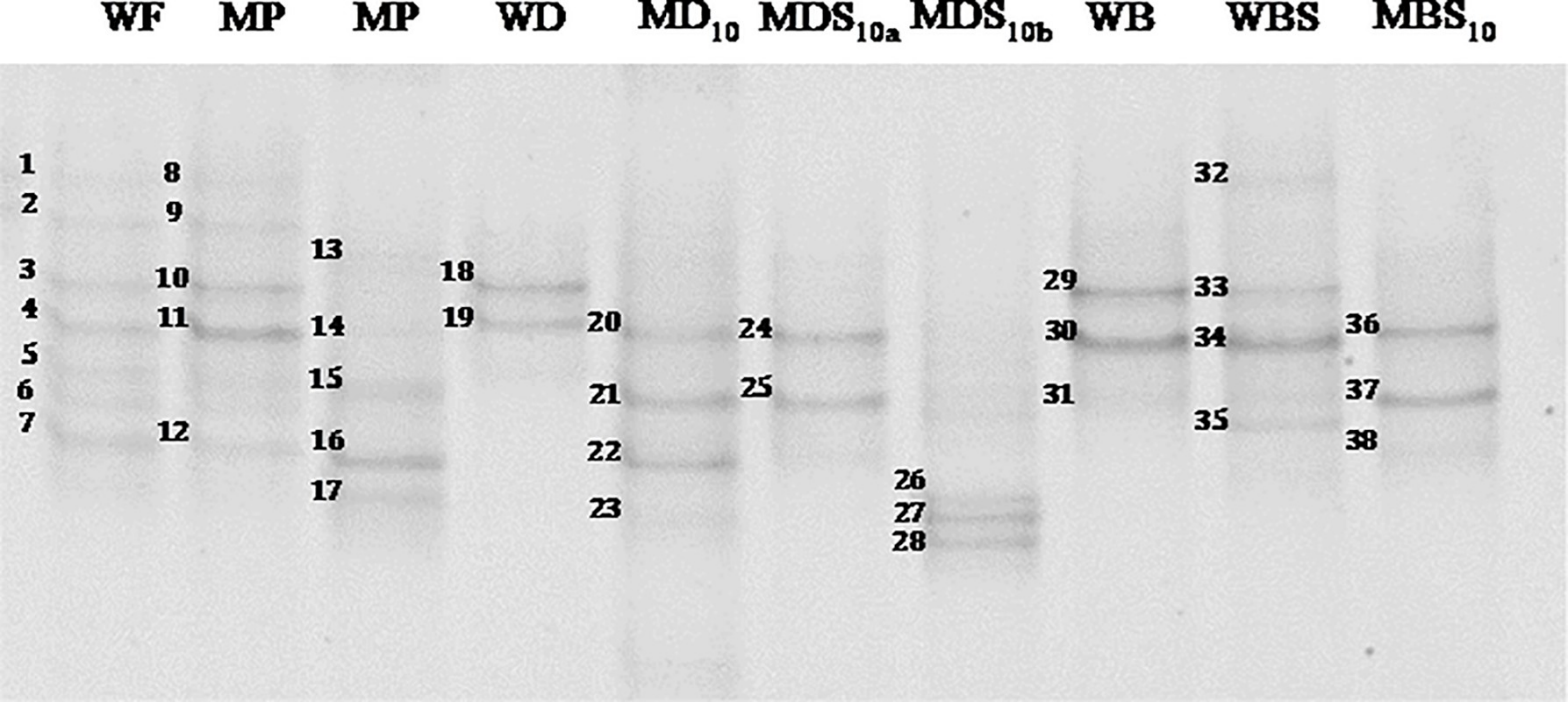
DGGE profiles of the DNA extracted form the spore-forming bacteria bulk cells washed off the PCA medium and amplified with primers 338fGC and 518r. The bands indicated by the numbers were excised, reamplified and subjected to sequencing. The identification of the bands is reported in Table 6. MDS_10a_ and MDS_10b_ correspond to the spore forming bacteria collected from the first and the second bread making trials, respectively. PCA, Plate Count Agar. MP, mealworm powder. Samples are codified as reported in Table 1.

Fig 4 shows DGGE profiles obtained from the microbial DNA extracted from the bulk cells; the closest relatives, the percent identities, and the accession number of sequences obtained from selected DGGE bands are reported in Table 6.

**Table 6:** Sequencing results from the bands cut from the denaturing gradient gel electrophoresis (DGGE) gels obtained from the amplified fragments of the DNA extracted directly from the colonies washed off the PCA plates for the spore counts.

Samples	Band code	Bands^a^	Closest relative	% Ident.^b^	Acc. No.^c^
WF		1	*Bacillus amyloliquefaciens*	99%	KJ603230
		2	*Bacillus amyloliquefaciens*	100%	KJ603230
		3	*Bacillus subtilis*	100%	KJ603239
		4	*Bacillus subtilis*	100%	KJ603239
	79E	**5**	*Bacillus sp*. *Bacillus amyloliquefaciens*	100% 99%	GQ169106 KJ603230
	79F	**6**	*Bacillus sp*. *Bacillus subtilis* *Bacillus amyloliquefaciens*	100% 100% 99%	GQ169106 KJ603239 KJ603230
		7	*Bacillus amyloliquefaciens*	99%	KJ603230
MP	83A	**8**	*Bacillus subtilis* *Bacillus vietnamensis* *Bacillus velezensis*	100% 100% 100%	KJ603239 LC325200 CP023320
	83B	**9**	*Bacillus amyloliquefaciens*	100%	KJ603230
	83C	**10**	*Bacillus subtilis*	100%	KJ603239
	83D	**11**	*Bacillus subtilis*	100%	KJ603239
	83E	**12**	*Bacillus amyloliquefaciens*	99%	KJ603230
	127A	**13**	*Bacillus sp*.	100%	GQ169106
	127B	**14**	*Paenibacillus sp*.	99%	MF139331
		15	*Bacillus sp*.	100%	GQ169106
		16	*Bacillus amyloliquefaciens*	99%	KJ603230
	127E	**17**	*Bacillus sp*. *Paenibacillus sp*.	100% 99%	GQ169106 MF139331
WD	135A	**18**	*Bacillus subtilis*	100%	KJ603239
		19	*Bacillus sp*.	100%	GQ169106
MD_10_		20	*Paenibacillus sp*.	99%	MF139331
		21	*Bacillus sp*.	100%	GQ169106
	133C	**22**	failed	-	-
	133D	**23**	*Bacillus sp*.	100%	GQ169106
MDS_10_	97A	24	*Paenibacillus sp*.	99%	MF139331
	97B	25	*Bacillus sp*.	100%	GQ169106
	131A	**26**	*Brevibacillus agri*	99%	KX783536
	131B	**27**	*Brevibacillus agri*	99%	KX783536
	131C	**28**	*Brevibacillus agri*	99%	KX783536
WB	139	29	*Bacillus subtilis*	100%	KJ603239
		30	*Bacillus subtilis*	100%	KJ603239
		31	*Bacillus sp*.	100%	GQ169106
WBS	123A	**32**	*Bacillus amyloliquefaciens*	99%	KJ603230
		33	*Bacillus subtilis*	100%	KJ603239
		34	*Bacillus subtilis*	100%	KJ603239
	123D	**35**	*Bacillus amyloliquefaciens*	99%	KJ603230
MBS_10_	137A	**36**	*Bacillus sp*.	100%	GQ169106
	137B	**37**	*Bacillus sp*.	100%	GQ169106
	137C	**38**	*Bacillus sp*.	100%	GQ169106

^a^ Selected bands excised from the agarose gels and subjected to sequencing are numbered.

^b^ Percentage of identical nucleotides in the sequence obtained from the DGGE bands and the sequence of the closest relative found in the GenBank database.

^c^ Accession number of the sequence of the closest relative found by a BLAST search. PCA, Plate Count Agar. MP, mealworm powder. Samples of doughs (D) admixed with baker’s yeast as leavening agents and sourdough (S) and experimental breads (B) are codified as reported in Table 1.

High percent identities, between 99 and 100%, were obtained for all of the sequenced bands. The most prevalent genus detected in all the samples belonged to the closest relatives to *Bacillus*. In more detail, the closest relatives to the spoilage species *Bacillus amyloliquefaciens* and *Bacillus subtilis* were broadly detected. These two *Bacillus* species, known to be the causative agent of the ropy spoilage of bread, have been frequently detected in raw materials used in bread making.

In such a context, the use of sourdough can represent a possible mitigation strategy. Indeed, as reported by Ziane et al. [61], low pH values prevent spores from germinating and can also exert an inhibitory activity against the recovery of injured spores. Based on the abovementioned available scientific literature, the assessment of bacterial spores that survive a heat treatment represents a pivotal activity for a proper risk assessment, which should also comprise the determination of spores from anaerobic spore-formers, such as *Clostridium* species. Valerio et al. [56] reported the presence of spoilage activity of strains of *B*. *amyloliquefaciens*, *B*. *subtilis* and *B*. *pumilus* in bread after heat treatment at 100°C for 10 min.

Though no microbiological criteria are set by Regulation 2073/2005 [62] as amended by Regulation 1441/2007 [63] for *Clostridium perfringens* and *Bacillus cereus* in ready-to-eat foods, including backed goods, aliquots of experimental breads fortified with mealworm powder were subjected to enumeration of both these spore forming human pathogens, which showed viable counts below 1 log cfu g^-1^.

### Amino acid composition

The amino acid contents of MP, WF and the different breads enriched with insect powder are summarized in Table 7.

**Table 7:** Amino acid composition (mg/100 mg fresh weight) of mealworm powder (M), wheat flour (W) and breads (B) produced with wheat flour or different blend of wheat flour and mealworm powder admixed with baker’s yeast and sourdough (S) as leaving agents.

Amino acids (mg/100 mg)	Samples							
	MP	WF	WB	MB_5_	MB_10_	WBS	MBS_5_	MBS_10_
*Essential*		*** ***						** **
His	1.23 ± 0.19	0.23 ± 0.03^a^	0.18 ± 0.02^a^	0.25 ± 0.02^a^	0.20 ± 0.05^a^	0.17 ± 0.02^a^	0.21 ± 0.01^a^	0.23 ± 0.01^a^
Thr	3.81 ± 0.45	0.67 ± 0.12^bc^	0.60 ± 0.08^bc^	0.70 ± 0.01^abc^	0.82 ± 0.05^ab^	0.57 ± 0.03^c^	0.71 ± 0.09^abc^	0.94 ± 0.06^a^
Tyr	2.58 ± 0.35	0.25 ± 0.04^b^	0.25 ± 0.01^b^	0.31 ± 0.02^b^	0.40 ± 0.05^a^	0.25 ± 0.01^b^	0.30 ± 0.00^b^	0.44 ± 0.03^a^
Val	0.43 ± 0.22	0.12 ± 0.01^a^	0.12 ± 0.01^a^	0.11 ± 0.01^a^	0.15 ± 0.04^a^	0.10 ± 0.01^a^	0.12 ± 0.02^a^	0.16 ± 0.04^a^
Met	3.20 ± 0.66	0.51 ± 0.07^bc^	0.49 ± 0.02^c^	0.57 ± 0.02^bc^	0.74 ± 0.04^a^	0.49 ± 0.02^c^	0.60 ± 0.03^b^	0.83 ± 0.01^a^
Phe	1.43 ± 0.20	0.53 ± 0.07^b^	0.47 ± 0.01^b^	0.52 ± 0.02^b^	0.57 ± 0.07^ab^	0.47 ± 0.02^b^	0.53 ± 0.01^b^	0.66 ± 0.01^a^
Iso	1.73 ± 0.25	0.35 ± 0.05^c^	0.32 ± 0.01^c^	0.38 ± 0.01^bc^	0.46 ± 0.05^ab^	0.32 ± 0.02^c^	0.38 ± 0.00^bc^	0.52 ± 0.01^a^
Leu	2.99 ± 0.50	0.75 ± 0.08^c^	0.68 ± 0.02^c^	0.79 ± 0.03^bc^	0.92 ± 0.08^ab^	0.67 ± 0.03^c^	0.80 ± 0.01^bc^	1.05 ± 0.02^a^
Lys	2.21 ± 0.22	0.20 ± 0.09^a^	0.13 ± 0.03^a^	0.21 ± 0.02^a^	0.21 ± 0.03^a^	0.14 ± 0.04^a^	0.25 ± 0.09^a^	0.27 ± 0.05^a^
Trp	0.44 ± 0.00	0.21 ± 0.01^a^	0.07 ± 0.03^c^	0.11 ± 0.03^bc^	0.14 ± 0.02^ab^	0.10 ± 0.02^bc^	0.11 ± 0.01^bc^	0.13 ± 0.01^abc^
**Total EAA**	**20.05 ± 3.05**	**3.83 ± 0.56**^**bc**^	**3.31 ± 0.12**^**c**^	**3.97 ± 0.13**^**bc**^	**4.61 ± 0.45**^**ab**^	**3.28 ± 0.15**^**c**^	**4.02 ± 0.20**^**bc**^	**5.24 ± 0.02**^**a**^
*Non essential*	*** ***	*** ***						** **
Asp + Asn	3.12 ± 0.45	0.43 ± 0.05^bcd^	0.40 ± 0.02^d^	0.52 ± 0.01^b^	0.71 ± 0.07^a^	0.41 ± 0.03^cd^	0.52 ± 0.00^bc^	0.80 ± 0.01^a^
Glu + Gln	4.22 ± 0.61	3.75 ± 0.42^ab^	3.38 ± 0.06^b^	3.41 ± 0.18^b^	3.68 ± 0.24^ab^	3.32 ± 0.19^b^	3.50 ± 0.16^bc^	4.29 ± 0.11^a^
Ser	1.79 ± 0.23	0.38 ± 0.05^b^	0.39 ± 0.02^b^	0.40 ± 0.04^b^	0.45 ± 0.04^ab^	0.38 ± 0.04^b^	0.40 ± 0.03^b^	0.58 ± 0.02^a^
Gly	1.28 ± 0.30	0.18 ± 0.01^c^	0.17 ± 0.01^c^	0.19 ± 0.00^c^	0.24 ± 0.01^b^	0.17 ± 0.01^c^	0.18 ± 0.01^c^	0.27 ± 0.01^a^
Arg	2.22 ± 0.36	0.37 ± 0.04^bc^	0.33 ± 0.02^c^	0.41 ± 0.02^b^	0.52 ± 0.02^a^	0.33 ± 0.01^c^	0.41 ± 0.02^b^	0.56 ± 0.00^a^
Ala	3.09 ± 0.55	0.32 ± 0.04^bc^	0.31 ± 0.02^bc^	0.40 ± 0.00^b^	0.52 ± 0.06^a^	0.30 ± 0.01^c^	0.40 ± 0.00^b^	0.60 ± 0.03^a^
Pro	3.50 ± 0.58	1.11 ± 0.02^bc^	1.20 ± 0.16^abc^	1.15 ± 0.03^abc^	1.55 ± 0.27^ab^	1.06 ± 0.12^c^	1.10 ± 0.00^bc^	1.61 ± 0.11^a^
**Total NEAA**	**19.21 ± 3.09**	**6.53 ± 0.63**^**bc**^	**6.17 ± 0.24**^**c**^	**6.49 ± 0.28**^**bc**^	**7.66 ± 0.71**^**ab**^	**5.98 ± 0.28**^**c**^	**6.52 ± 0.13**^**bc**^	**8.71 ± 0.02**^**a**^
**Total AA**	**39.27 ± 6.13**	**10.37 ± 1.19**^**bc**^	**9.48 ± 0.33**^**c**^	**10.45 ± 0.41**^**bc**^	**12.28 ± 1.16**^**ab**^	**9.25 ± 0.39**^**c**^	**10.54 ± 0.33**^**bc**^	**13.95 ± 0.00**^**a**^

Means ± standard deviations of triplicate independent experiments are shown.

Values in the same row with different letters (a, b, c, d) as superscripts are significantly different (P < 0.05).

Comparison of the WF with control breads prepared with and without sourdough revealed that all amino acids were present at the same level, with the exception of tryptophan, which was significantly lower in control breads. This result is in accordance with the finding that both the dough fermentation and the backing procedure can reduce the tryptophan content in breads [64, 65]. Moreover, Morales et al. [66] reported that tryptophan degradation in cookie model systems depends on both temperature and time.

All amino acids, with the exception of glutamate, were present at higher levels in the MP compared to WF. Accordingly, breads containing MP exhibited increases in both essential and nonessential amino acids that were statistically significant in breads enriched with 10% MP (MB_10_ and MBS_10_) in comparison to control breads (WB and WBS). Among the essential amino acids, tyrosine, methionine, isoleucine, and leucine exhibited the highest average increases in breads fortified with 10% MP, containing 68%, 60%, 53% and 46%, respectively.

The MP also contained more total free amino acids compared to WF, and accordingly, breads enriched with 10% MP exhibited a significant increase in free amino acids (Table 8).

**Table 8:** Free amino acid composition (mg/100 g fresh weight) of mealworm powder (M), wheat flour (W) and breads (B) produced with wheat flour or wheat flour and 10% mealworm powder admixed with baker’s yeast and sourdough (S) as leaving agents.

Free aminoacids (mg/100 g)	Samples					
	MP	WF	WB	MB_10_	WBS	MBS_10_
His	47.84 ± 2.65	1.14 ± 0.11	4.11 ± 0.96	15.81 ± 2.62	3.21 ± 1.77	12.16 ± 0.84
Thr	109.36 ± 9.63	2.6 ± 0.37	4.22 ± 1.72	13.21 ± 2.65	3.27 ± 0.11	10.25 ± 1.30
Tyr	288.85 ± 20.30	1.58 ± 0.20	1.41 ± 0.76	27.6 ± 4.31	2.31 ± 1.23	25.19 ± 0.44
Val	16.36 ± 2.16	0.29 ± 0.01	0.14 ± 0.19	2.36 ± 0.89	0.5 ± 0.29	2.83 ± 0.33
Met	159.28 ± 23.21	1.77 ± 0.22	1.79 ± 0.10	20.49 ± 1.01	3.4 ± 1.23	18.35 ± 0.13
Phe	48.30 ± 8.69	1.12 ± 0.08	1.13 ± 0.22	5.46 ± 0.58	2.32 ± 0.14	6.61 ± 1.79
Iso	88.73 ± 13.71	0.87 ± 0.11	0.68 ± 0.20	7.62 ± 1.35	1.48 ± 0.13	7.72 ± 0.17
Leu	92.51 ± 9.33	1.07 ± 0.16	1.09 ± 0.24	7.31 ± 1.32	2.8 ± 0.12	10.1 ± 0.28
Lys	131.53 ± 0.78	n.d.	3.87 ± 0.54	9.47 ± 0.44	6.46 ± 1.03	10.18 ± 1.69
Asp + Asn	37.34 ± 3.01	15.42 ± 5.11	6.03 ± 4.64	15.02 ± 3.12	7.14 ± 5.27	18.65 ± 1.20
Glu + Gln	183.98 ± 16.46	8.45 ± 1.62	8.65 ± 1.26	26.44 ± 6.21	7.71 ± 1.07	25.02 ± 2.65
Ser	49.50 ± 5.83	2.16 ± 0.56	1.9 ± 0.23	5.28 ± 0.95	1.87 ± 0.09	7.02 ± 0.48
Gly	77.28 ± 12.08	0.78 ± 0.03	1.97 ± 0.10	7.83 ± 0.60	2.41 ± 0.35	8.67 ± 0.04
Arg	391.50 ± 35.55	4.17 ± 0.42	6.72 ± 2.19	35.09 ± 7.72	7.74 ± 1.89	35.64 ± 1.28
Ala	172.90 ± 6.65	3.45 ± 0.53	3.91 ± 1.24	19.15 ± 3.23	5.41 ± 0.87	23.43 ± 0.45
Pro	592.467 ± 93.71	0.74 ± 0.79	0.19 ± 0.08	22.26 ± 10.25	0.35 ± 0.09	33.36 ± 3.10
**Total AA**	**2487.72 ± 59.18**	**45.61 ± 10.33**	**47.83 ± 0.16**	**240.41 ± 42.26**	**58.39 ± 7.95**	**255.21 ± 15.42**

n.d. not detectable

Means ± standard deviations of triplicate independent experiments are shown.

Addition of sourdough slightly increased the free amino acid content in breads prepared with or without the MP, likely reflecting the contribution of the proteolytic activity of the dough’s microbiota. By inspecting the individual amino acid content, all analyzed amino acids were markedly increased in the breads, but to a different extent depending on the use of the sourdough.

It has been established that at certain concentrations, the presence of free asparagine and reducing sugars can increase the content of acrylamide during heating of food products [67]. Under our conditions of acidic hydrolysis, asparagine is completely hydrolyzed to aspartic acid, and we observed a marked increase of free aspartic acid in breads enriched with 10% MP. Since acrylamide is classified as a probable carcinogen in humans [68], further studies are surely needed to investigate its content in enriched breads.

### Total fatty acid composition

The total FA composition of the raw ingredients (WF and MP) (Table 9) was in accordance with previously published data [33, 69].

**Table 9:** Total fatty acid composition (% w/w; mean ± SD of two replicates, calculated by the GC peak area normalization method) of wheat flour, mealworm powder and bread samples.

Fatty Acid	Samples							
	MP	WF	WB	WBS	MB_5_	MBS_5_	MB_10_	MBS_10_
C12:0 (lauric)	0.34 ± 0.03^a^	0.01 ± 0.00^e^	0.06 ± 0.01^de^	0.03 ± 0.00^e^	0.15 ± 0.06^cd^	0.21 ± 0.03^bc^	0.28 ± 0.02^ab^	0.28 ± 0.03^ab^
C14:0 (myristic)	3.10 ± 0.08^a^	0.12 ± 0.01^d^	0.18 ± 0.02^d^	0.12 ± 0.01^d^	1.45 ± 0.14^c^	1.43 ± 0.06^c^	1.90 ± 0.05^b^	1.85 ± 0.12^b^
C15:0 (pentadecanoic)	0.17 ± 0.00^a^	0.14 ± 0.06^a^	0.11 ± 0.02^a^	0.10 ± 0.01^a^	0.11 ± 0.00^a^	0.11 ± 0.01^a^	0.12 ± 0.02^a^	0.13 ± 0.00^a^
C16:0 (palmitic)	21.55 ± 0.20^a^	18.29 ± 0.46^abc^	19.37 ± 1.90^ab^	15.12 ± 0.13^c^	16.17 ± 0.18^bc^	16.09 ± 0.37^c^	16.49 ± 0.10^bc^	16.35 ± 0.16^bc^
C16:1 (palmitoleic)	1.68 ± 0.01^a^	0.17 ± 0.00^c^	1.69 ± 0.17^b^	1.14 ± 0.11^a^	1.62 ± 0.03^a^	1.69 ± 0.04^a^	1.72 ± 0.00^a^	1.71 ± 0.09^a^
C17:0 (margaric)	0.17 ± 0.05^a^	0.10 ± 0.01^ab^	0.10 ± 0.02^ab^	0.07 ± 0.01^b^	0.12 ± 0.01^ab^	0.12 ± 0.00^ab^	0.14 ± 0.01^ab^	0.13 ± 0.00^ab^
C18:0 (stearic)	3.72 ± 0.03^a^	0.87 ± 0.04^d^	0.90 ± 0.15^d^	0.79 ± 0.08^d^	1.95 ± 0.08^c^	1.88 ± 0.15^c^	2.36 ± 0.04^b^	2.24 ± 0.11^bc^
C18:1Δ9 (oleic)	36.79 ± 0.35^a^	9.05 ± 0.05^d^	8.02 ± 0.29^d^	8.49 ± 0.11^d^	24.38 ± 2.17^bc^	23.53 ± 2.73^c^	28.30 ± 0.13^b^	27.68 ± 0.65^bc^
C18:1Δ11 (vaccenic)	0.34 ± 0.06^d^	0.80 ± 0.01^a^	0.70 ± 0.02^b^	0.75 ± 0.03^ab^	0.52 ± 0.01^c^	0.53 ± 0.00^c^	0.45 ± 0.01^c^	0.46 ± 0.01^c^
C18:2Δ9,12 (linoleic)	29.65 ± 0.08^c^	65.71 ± 0.45^a^	64.31 ± 1.70^a^	68.38 ± 0.58^a^	49.90 ± 2.33^b^	50,60 ± 2.91^b^	44.98 ± 0.16^b^	45.84 ± 0.59^b^
C18:3Δ9,12,15 (linolenic)	1.85 ± 0.02^d^	3.62 ± 0.14^ab^	3.89 ± 0.19^a^	4.07 ± 0.01^a^	3.07 ± 0.13^bc^	3.15 ±0.30^bc^	2.80 ± 0.03^c^	2.91 ± 0.11^c^
C20:1Δ11 (eicosenoic)	0.10 ± 0.00^c^	0.33 ± 0.02^a^	0.20 ± 0.06^bc^	0.29 ± 0.03^ab^	0.12 ± 0.03^c^	0.17 ± 0.01^c^	0.13 ± 0.01^c^	0.14 ± 0.01^c^
C20:2Δ11,14 (eicosadienoic)	0.09 ± 0.01^a^	0.09 ± 0.01^a^	0.06 ± 0.03^a^	0.09 ± 0.01^a^	0.08 ± 0.00^a^	0.08 ± 0.01^c^	0.08 ± 0.00^a^	0.08 ± 0.00^a^
OP	0.48 ± 0.06	0.74 ± 0.08	0.39 ± 0.12	0.58 ± 0.30	0.38 ± 0.16	0.44 ± 0.04	0.28 ± 0.06	0.22 ± 0.01
SFA	29.05 ± 0.39^a^	19.51 ± 0.48^bc^	20.73 ± 1.87^b^	16.22 ± 0.23^c^	19.94 ± 0.46^b^	19.83 ± 0.54^b^	21.27 ± 0.09^b^	20.98± 0.12^b^
MUFA	38.91 ± 0.39^a^	10.34 ± 0.08^d^	10.62 ± 0.23^d^	10.67 ± 0.06^d^	26.64 ± 2.16^bc^	25.91 ± 2.70^c^	30.60 ± 0.15^b^	29.99 ± 0.59^bc^
PUFA	31.58 ± 0.06^c^	69.41 ± 0.31^a^	68.26 ± 1.84^a^	72.53 ± 0.58^a^	53.05 ± 2.46^b^	53.82 ± 3.20^b^	47.86 ± 0.18^b^	48.83 ± 0.70^b^
n-6/n-3	16.07 ± 0.23^b^	18.17 ± 0.83^a^	16.57 ± 0.71^ab^	16.82 ± 0.11^ab^	16.25 ± 0.09^ab^	16.09 ± 0.59^b^	16.06 ± 0.11^b^	15.79 ± 0.37^b^
AI	0.49 ± 0.01^a^	0.24 ± 0.01^cd^	0.26 ± 0.03^bc^	0.19 ± 0.00^d^	0.28 ± 0.01^bc^	0.28 ± 0.01^bc^	0.31 ± 0.00^b^	0.30 ± 0.00^b^
TI	0.71 ± 0.01^a^	0.39 ± 0.01^bc^	0.42 ± 0.05^b^	0.31 ± 0.01^c^	0.41 ± 0.01^b^	0.41 ± 0.02^bc^	0.45 ± 0.00^b^	0.44 ± 0.01^b^
h/H	2.77 ± 0.05^c^	4.27 ± 0.13^b^	3.93 ± 0.49^b^	5.32 ± 0.07^a^	4.40 ± 0.10^ab^	4.42 ± 0.14^ab^	4.14 ± 0.01^b^	4.20 ± 0.01^b^

Abbreviations: SFA, saturated fatty acids; MUFA, monounsaturated fatty acids; PUFA, polyunsaturated fatty acids; OP, Other unidentified Peaks; SD, standard deviation; Cm:n Δx, m = number of carbon atoms, n = number of double bonds, x = position of double bonds; AI, atherogenic index = (C12:0 + 4 × C14:0 + C16:0)/(Σn-6 + Σn-3 + ΣMUFA); TI, thrombogenic index = (C14:0 + C16:0 + C18:0)/[0.5 × ΣMUFA + 0.5 × Σn-6 + 3 × Σn-3 + (Σn-3/Σn-6)]; h/H, hypocholesterolemic/hypercholesterolemic ratio = (C18:1 + C18:2 + C18:3 + C20:2 + C20:4 + C20:5 + C22:5 + C22:6)/(C14:0 + C16:0).

Comparison for all pairs using Turkey-Kramer HSD, Alpha = 0.05. Means in the same row bearing different letters (a, b, c, d, e) differ significantly (P < 0.05).

The five most-represented FAs (palmitic, stearic, oleic, linoleic, and linolenic) accounted for 94% and 97% of total FAs in MP and WF, respectively. In MP, the percentages of the above mentioned FAs were consistent with the values reported by Barroso et al. [70] for the same insect species. The relative percentages of saturated fatty acids (SFA) (mainly palmitic and stearic) and monounsaturated fatty acids (MUFA) (mainly oleic) were significantly higher in MP than WF, whereas WF was characterized by higher levels of polyunsaturated fatty acids (PUFA) (mainly linoleic and linolenic). Therefore, the FA profile of MP seemed less sensitive to oxidation than WF lipids, during both storage of raw material and baking.

As expected, no significant differences in FA composition between WF and control breads (WB, WBS) were observed. Myristic, stearic and oleic acid levels determined the main changes in FA composition of breads produced with the tested blends (WF + MP). However, neither the MP inclusion level (5% and 10%, on weight basis) nor the bread-making procedure (with or without sourdough) significantly affected the FA composition of the final products. The comparison between the control breads (WB and WBS) and the fortified breads (MB_5_, MBS_5_, MB_10_, MBS_10_) reflected the FA profiles of the raw materials. MB breads exhibited higher percentages of SFA (mainly stearic acid) and MUFA (mainly oleic acid) and lower relative percentages of PUFA (mainly linoleic and linolenic acids) than WF breads.

The nutritional quality of the lipid fraction of breads and raw materials was assessed by calculating the PUFA n-6/n-3 ratio, Atherogenic Index (AI), Thrombogenic Index (TI), and hypocholesterolemic/Hypercholesterolemic ratio (h/H) [3]. Significant differences between the nutritional properties of MP and WF lipids were observed, with the former being characterized by lower n-6/n-3 and h/H ratios and higher AI and TI indexes than the latter. According to nutritionists, the n-6/n-3 ratio should range from 1:1 to 5:1, whereas a ratio of 10:1 has adverse consequences [71]. The lipid fractions of raw materials and breads were hence characterized by too-high n-6/n-3 ratio values. All samples analyzed exhibited AI and TI values less than 1.00, which is considered to be the appropriate value of those indexes for a healthy dietary lipid. No significant differences in nutritional quality of lipids were observed between fortified and control breads.

### Color analysis

Color is a key property of baked products, since, together with volume and texture, it influences consumer acceptance. Data of color assessment with the CIELAB system on crust and crumb of both fortified and control breads are presented in Table 10.

**Table 10:** Effect of mealworm powder addition on color parameter of experimental and control breads.

Samples	Crust			Crumb		
	L*	a*	b*	L*	a*	b*
WB	77.38 ± 1.33^a^	-1.54 ± 1.15^c^	23.01 ± 1.37^c^	71.78 ± 0.53^a^	-5.19 ± 0.08^d^	20.77 ± 0.51^ab^
WBS	71.80 ± 1.63^b^	2.56 ± 1.12^ab^	27.30 ± 2.28^ab^	74.52 ± 0.22^a^	-5.46 ± 0.12^d^	19.95 ± 0.09^ab^
MB_5_	70.58 ± 1.11^b^	0.58 ± 0.93^bc^	23.58 ± 1.25^bc^	63.86 ± 1.22^b^	-3.12 ± 0.17^c^	19.38 ± 0.64^b^
MBS_5_	69.99 ± 1.74^b^	0.66 ± 0.76^bc^	24.51 ± 1.49^abc^	64.24 ± 0.69^b^	-2.40 ± 0.23^b^	21.17 ± 0.69^a^
MB_10_	62.61 ± 1.87^c^	5.13 ± 0.58^a^	27.98 ± 0.28^a^	63.22 ± 1.86^b^	-1.96 ± 0.54^ab^	20.21 ± 0.99^ab^
MBS_10_	64.49 ± 0.77^c^	3.49 ± 0.21^a^	27.63 ± 1.13^a^	61.84 ± 0.95^b^	-1.62 ± 0.14^a^	20.39 ± 0.61^ab^

L*, lightness; a*, redness; b*, yellowness

Means in column followed by different letters (a, b, c, d) are significantly different (*P*<0.05)

Samples are codified as reported in Table 1.

As a general trend, fortification with increasing amounts of MP shows a reduction of crust luminosity (L*) as well as an increase of redness (a*) and of yellowness (b*) of crust. As far as the crumb is concerned, a less definite correspondence emerged between the level of substitution with MP and change of the CIELAB coordinates.

In baked goods, the change of color during the baking process is mainly dependent on either the cooking time and temperature or the color of the raw materials. For the crust, the change in color is specifically dependent on the Maillard reaction, which takes place during baking, between amine groups (e.g. amino acids and proteins) and carbonyl compounds (e.g. reducing sugars). As it emerges from Table 10, the higher the level of substitution of wheat flour with MP, the more brownish the crust was, thus supporting the highest values of a* and b* found in crust of breads fortified with 10% mealworm powder. These findings agree with those recently collected by González et al. [13] on crumb of breads produced with various species of edible insects, including *T*. *molitor*. Moreover, an enhancement of the Maillard reaction on the crust of breads fortified with 10% of MP might be hypothesized on the basis of the significantly higher content in amino acids and proteins of MP in respect with WF.

Overall, the type of leavening agent used (sourdough plus baker’s yeast or the sole baker’s yeast) did not affect the assessed color parameters in bread crumb. Intriguingly, a significant decrease of L* and an increase of a* and b* was seen in the crust of control breads containing sourdough in comparison to the sole baker’s yeast. This latter finding might feasibly be explained by the proteolytic activity of sourdough lactobacilli responsible for a high amino acid release during sourdough fermentation [72].

### Sensory analysis

Consumer acceptability is undoubtedly a further key parameter for launching a new food product into the market. The results of sensory evaluation of the fortified breads containing different levels of MP substitution compared to the controls are shown in Table 11.

**Table 11:** Overall liking of experimental breads (B) produced with wheat flour and different blends of wheat flour (W) and mealworm powder (M) admixed with sourdough (S) and baker’s yeast as leavening agents.

	Experimental bread					
	WB	WBS	MB_5_	MSB_5_	MB_10_	MSB_10_
Overall liking	7.7^a^	7.9^a^	6.5^b^	6.3^b^	6.2^b^	6.0^b^

The degree of overall liking was ranked in accordance with a 9-point hedonic scale ranging from 1 (dislike extremely) to 9 (like extremely)

Samples are codified as reported in Table 1

Average values of independent experiments are shown.

Within each row, means followed by different letters (a, b) are significantly different (P < 0.05).

Overall a significantly highest liking for control breads in respect with fortified breads was seen, irrespective of the type of leavening agent used. Moreover, for the latter breads, no differences were seen in the overall liking, irrespective of the amount of MP added. If these data are compared with those collected by Osimani et al. [3] in a similar study, where the effect of cricket powder on sensory properties of fortified breads was assessed, a higher acceptability was scored by mealworm breads in comparison to cricket breads. This latter finding might be explained by the occurrence of differences in the sensory features of the two insect-based powders used in bread-making, namely color, flavor and taste. With regards to the latter two parameters, as recently elucidated by various authors [73, 74], different insect species are characterized by quite different tastes and flavor, depending on a number of factors, such as: (i) pheromones occurring at the insect surface; (ii) type of feed; (iii) presence of an exoskeleton.

Mealworm powder is produced from larvae typically fed on cereal bran or flour and is characterized by a sweet, almost nutty flavor; a nutty, cocoa smell; and a light to medium brown color. By contrast, cricket powder is produced from adult insects, whose external anatomy is made up of the exoskeleton, head, eyes, mandibles, antennae, legs and wings. Crickets are generally fed with grass; the powder from this insect species is characterized by: a strong crustacean-like, cooked legumes-like and earthy aroma and a medium to dark brown color with some coarse particulates visible, deriving from insect exoskeleton parts. The exoskeleton is made of proteins and chitin, whose digestibility in humans has long be debated and denied [75]. Given these premises, either the texture or the strong aroma and smell of cricket powder might have contributed to make the corresponding fortified breads less attractive than those assayed in the present study.

### Multivariate analysis

**Fig 5:**
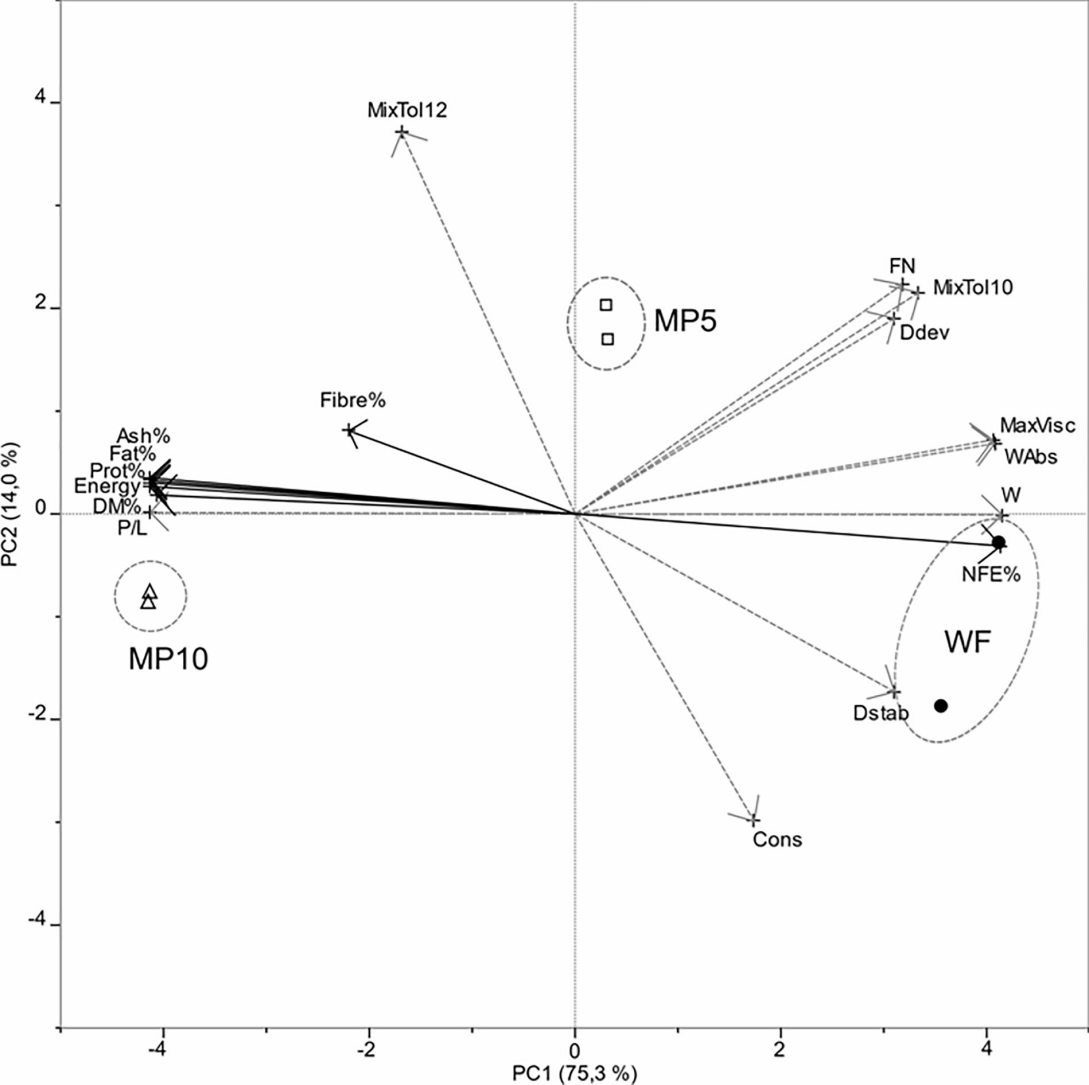
Biplot of PCA scores of wheat flour samples (WF) and its blends with mealworm powder (MP_5_, MP_10_) and loadings of variables. Analytical characteristics are in continuous black vectors and rheological parameters are in dashed grey vectors. Samples are codified as reported in Table 1.

The relationships between chemical composition of WF and WF/MP blends and the visco-elastic properties of their doughs (Alveograph and Farinograph data) were assessed via PCA (Fig 5).

The contribution of MP lipids (27.69%), nongluten proteins (51.71%), and minerals (3.65%) to the chemical composition of blends clearly affected both the chemical and functional properties of the same. The analytical variables significantly loaded on PC1, which explained 75.3% of total variance: a cluster of strongly positively correlated (vectors oriented in the same direction) composition parameters (Protein%, Fat%, Ash%, DM%, Energy) had negative loadings on PC1, whereas NFE% had a high positive loading. A significant negative correlation (variables positioned in opposite direction with respect to the axes origin and far from the plot origin) was observed between the cluster W (dough strength), WAbs (percentage of water absorption to yield dough of 500 BU consistency), and MaxVisc (maximum dough viscosity), with positive loadings on PC1, and P/L configuration ratio of the Alveograph, with a negative loading on PC1. PC2, which raised the total explained variance from 75.3% to 89.0%, was only affected by functional properties of flours, mainly by the mixing tolerance index after 12 min (MixTol12), falling number (FN) (positive loadings), and farinograph maximum consistency (Cons) (negative loading).

The sample scores determined the distribution of the samples on the plane defined by PC1 and PC2 (Fig 5). In a recent study concerning the use of cricket powder in bread making [3], we used a typical W300-type flour (“strong” flour), characterized by a P/L ratio of 0.9, a high protein content (14.21 ± 0.08%), and an FN value near 400. In this research study, WF was characterized by lower strength (W = 256–260) and protein content (11.38%), thus resulting in a lower water absorption percentage (59.6) and P/L ratio (0.65–0.68) (“medium strength” flour). However, the FN was higher (417–420), indicating a very low enzyme activity and very sound wheat quality. As expected, partial substitution of WF with increasing percentages of MP shifted the sample scores towards lower values along PC1. Those changes in chemical composition drove the differentiation of WF away from that of the blends, alongside an increase in the P/L ratio and decreases in W, WAbs, and MaxVisc. Interestingly, Cons and MixTol12 exhibited a nonlinear behavior, as the MP percentage increased in blends; higher intensities of Cons decreases and MixTol12 increases pulled the MP_5_ samples towards positive scores on PC2 and MP_10_ samples towards negative scores on PC2. It was also noticeable that the cluster of rheological variables Ddev, MixTol10, and FN significantly changed (decreased) only for MP_10_ samples, thus contributing to differentiation between MP_5_ samples (positive scores on PC2) and MP_10_ samples (negative scores on PC2).

**Fig 6:**
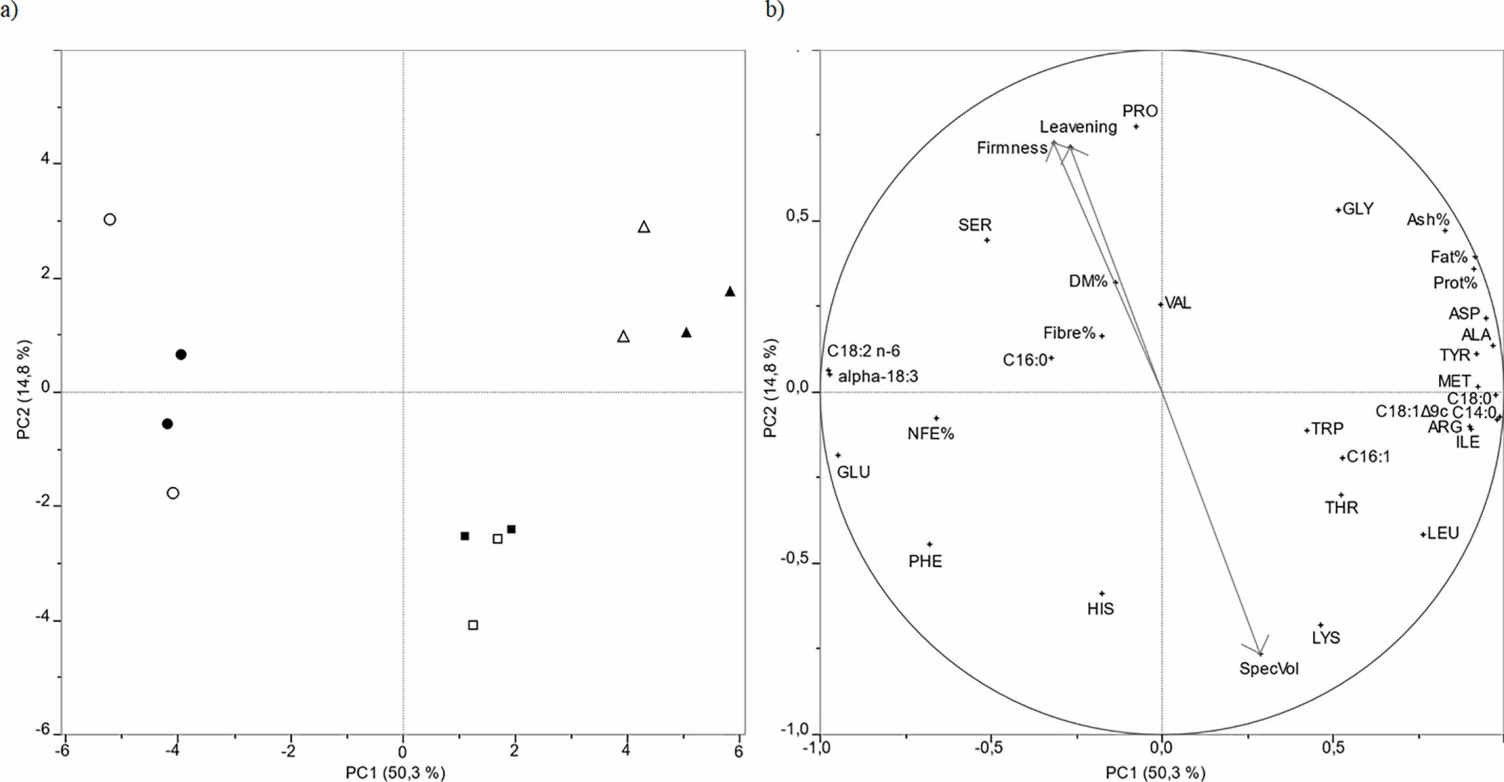
**PCA scores plot of bread samples (panel a) and PCA loadings plot of variables (panel b). Loading vectors of technological variables (Specific volume, Leavening, Firmness) are highlighted.** Amino acid composition data were expressed as relative percentages. Samples: ● = WB, ○ = WBS; _▀_ = MB_5_; □ = MBS_5_;▲ = MB_10_; Δ = MBS_10_. Samples are codified as reported in Table 1.

Experimental data about breads (proximate composition, fatty acid and amino acid compositions, and technological parameters) were also explored by means of PCA to evaluate the relationships between the structure of variables and objects (i.e., bread samples) (Fig 6).

PC1 was able to differentiate between WF breads and breads produced with the two blends. The former had positive relationships with variables having negative loadings in PC1 (mainly PUFA C18:2 and C18:3, and GLU), whereas SFA (C14:0, C18:0), MUFA (C18:1), a cluster of essential (MET, TYR, ILE, LEU) and nonessential (ALA, ASP, ARG) amino acids, Fat%, Protein% and Ash% pulled breads containing MP towards positive loadings on PC1. PC2 was useful for differentiating between the two levels of inclusion of MP in the two blends. In more detail, higher SpecVol and higher HIS and LYS relative percentages pulled the MP_5_ breads down to PC2 negative scores, whereas higher firmness and leavening percentages, together with higher PRO percentages, pulled the MP_10_ breads up to PC2 positive scores. Adding MP reduced bread firmness and dough leavening percentages (positive loadings on PC2) and increased specific volume of loaves (negative loadings on PC2), to a greater extent for MD_5_ and MDS_5_ than for MD_10_ and MDS_10_. The details of the bread making procedure (with or without SD) did not significantly affect either the chemical composition or technological properties of the final products. Indeed, loaves produced with the two blends lay undifferentiatedly on the positive PC2 space (MB_10_ and MBS_10_) and on the negative PC2 space (MB_5_ and MBS_5_), based on the MP substitution level.

Based on the above evidences, PCA permitted to differentiate samples on the basis of the substitution level of MP used for bread-making; furthermore, it allowed to highlight data structure as determined by internal data variance. More specifically, some essential (MET, TYR, ILE, LEU) and nonessential (ALA, ASP, ARG) amino acids as well as crude protein content (Prot%) were among the major sources of variability in data structure. Firmness, leavening and specific volume, as well as PRO, HIS, and LYS content represented a secondary source of variability.

## Conclusions and future perspectives

Among edible insects, mealworm larvae are easy to rear and can provide protein of high nutritional value [76, 77]. In some EU countries (e.g., the Netherlands), the farming of mealworms is already a profitable activity that can benefit from innovative exploitation processes [17]. In the present study, proof of concept was provided for the inclusion of MP into bread doughs leavened with sourdough and/or baker’s yeast. Results overall collected indicated that bread fortification with MP at the 5 and 10% of the soft wheat flour amount significantly affected breads properties depending on fortification level. As a general trend, an improvement of the protein and amino acid contents of the fortified breads was seen. Moreover, MP contributed to the improvements in both bread volume and softness, likely due to its lipid fraction. At this regard, MP appears as an adequate ingredient for bread fortification. Whereas technically, there are no major limitations to incorporate MP into bread dough at the assayed levels (5 and 10%), some considerations regarding microbial and sensory quality traits of bread enriched with mealworm powder can now be made. First, acceptability of fortified breads was negatively affected by comparison with control breads produced with the sole soft wheat flour. However, if overall liking scores collected in the present study were compared with those of a previous research from the same laboratory on breads fortified with cricket powder (at 10 and 30% substitution level), mealworm breads were characterized by a higher acceptability, feasibly due to the acknowledged differences in flavor and aroma of mealworm and cricket powders. Concerning the microbial quality, the occurrence of spore-forming bacteria in both doughs and breads fortified with mealworm powder highlighted potential spoilage and even human safety issues that still need to be resolved. At this regards, in future studies, it might be valuable to investigate the effect of strategies to reduce the amount of endospores in bread and, more in general, in baked leavened goods, such as the use of preservatives, e.g. propionate [78] or mitigation technologies, e.g. dehydration [78] or toasting [79].

Based on the Technology Readiness Level (TRL) scale, which characterizes the progress in the development of a technology from the idea (level 1) to the full deployment of the product in the marketplace (level 9), the proposed bread making technology can be situated at level 4 (validation in laboratory environment), which means that prototypes are ready to be tested in a simulated environment. Regarding implementation at the industrial scale, insect powders (including those from mealworms) are currently produced in Europe at the commercial scale. Moreover, in November 2017, a Finnish bakery launched the world’s first cricket-based bread to be offered to consumers in stores, thus suggesting that the production of breads with MP might easily be scaled up at the industrial scale.
